# Jamaican fruit bat (*Artibeus jamaicensis*) insusceptibility to mucosal inoculation with SARS-CoV-2 Delta variant is not caused by receptor compatibility

**DOI:** 10.1038/s44298-024-00037-1

**Published:** 2024-07-16

**Authors:** Julia R. Port, Jade C. Riopelle, Sarah van Tol, Arthur Wickenhagen, Eric Bohrnsen, Daniel E. Sturdevant, Rebecca Rosenke, Jamie Lovaglio, Justin Lack, Sarah L. Anzick, Kathleen Cordova, Kwe Claude Yinda, Patrick W. Hanley, Tony Schountz, Lon V. Kendall, Carl I. Shaia, Greg Saturday, Craig Martens, Benjamin Schwarz, Vincent J. Munster

**Affiliations:** 1 Laboratory of Virology, Division of Intramural Research, National Institute of Allergy and Infectious Diseases, National Institutes of Health, Hamilton, MT USA; 2 Research Technologies Branch, Division of Intramural Research, National Institute of Allergy and Infectious Diseases, National Institutes of Health, Hamilton, MT USA; 3 Rocky Mountain Veterinary Branch, Division of Intramural Research, National Institute of Allergy and Infectious Diseases, National Institutes of Health, Hamilton, MT USA; 4 Department of Microbiology, Immunology, and Pathology, Colorado State University, Fort Collins, CO USA

**Keywords:** Microbiology, Virology, SARS-CoV-2

## Abstract

The ancestral sarbecovirus giving rise to SARS-CoV-2 is posited to have originated in bats. While SARS-CoV-2 causes asymptomatic to severe respiratory disease in humans, little is known about the biology, virus tropism, and immunity of SARS-CoV-2-like sarbecoviruses in bats. SARS-CoV-2 has been shown to infect multiple mammalian species, including various rodent species, non-human primates, and Egyptian fruit bats. We show that SARS-CoV-2 can utilize Jamaican fruit bat (*Artibeus jamaicensis)* ACE2 spike for entry in vitro. Therefore, we investigate the Jamaican fruit bat as a possible in vivo model to study reservoir responses. We find that SARS-CoV-2 Delta does not efficiently replicate in Jamaican fruit bats in vivo. We observe infectious viruses in the lungs of only one animal on day 1 post-inoculation and find no evidence of shedding or seroconversion. This is possibly due to host factors restricting virus egress after aborted replication. Furthermore, we observe no significant immune gene expression changes in the respiratory tract but do observe changes in the intestinal metabolome after inoculation. This suggests that, despite its broad host range, SARS-CoV-2 is unable to infect all bat species, and Jamaican fruit bats are not an appropriate model to study SARS-CoV-2 reservoir infection.

## Introduction

SARS-CoV-2 has a posited ancestral origin in insectivorous Old World horseshoe bats (*Rhinolophus* spp.)^1,2^. While multiple animal models of human virus-host interactions and pathogenesis for COVID-19 exist^3^, little is known about the interaction of the virus with its putative bat reservoir. To understand how sarbecoviruses interact with their bat reservoirs, dedicated bat experimental in vitro, ex vivo, and in vivo models are required in which infection can be studied under controlled laboratory conditions and through which critical knowledge gaps regarding bat immunity and pathogenesis can be filled^4^. Due to the wide cross-species susceptibility to SARS-CoV-2^5,6^, it can be hypothesized that multiple bat species could be susceptible to infection and serve as potential models^7–9^.

It is technically and logistically challenging to use many bat species for laboratory studies, and laboratory colonies are very rare. However, different groups have successfully investigated coronavirus infections in bats. After inoculation of Leschenault’s rosette bats with a homogenate of betacoronavirus (clade b)-positive intestinal tissues, viral RNA was detected in fecal samples and intestinal tissue in the absence of clinical signs^10^. Intranasal and intraperitoneal inoculation of Jamaican fruit bat (*Artibeus jamaicensis*) with Middle East respiratory syndrome coronavirus (MERS)-CoV led to shedding of viral RNA in oral and rectal swabs, as well as viral RNA detection in multiple tissues, again in the absence of clinical signs^11^. However, WIV-1-CoV was unable to cause a robust infection in Egyptian fruit bats (*Rousettus aegyptiacus*)^12^. Multiple infection studies have examined the susceptibility of different bat species to SARS-CoV-2 infection, including Egyptian fruit bats^13^, insectivorous big brown bats (*Eptesicus fuscus*)^14^, and Brazilian free-tailed bats (*Tadarida brasiliensis*)^15,16^. While Egyptian fruit bats demonstrated detection of virus in the respiratory tract for several days in the absence of clinical disease, no virus replication was shown in big brown bats, and the susceptibility of Brazilian free-tailed bats requires further investigation.

Jamaican fruit bats breed well in captivity and harbor diverse coronaviruses in the wild^17^ and are susceptible to a broad range of viruses after experimental inoculation. This includes Tacaribe virus and rabies virus infections of varying severity depending on inoculation dose and virus strain, respectively, asymptomatic Zika virus infection, and clinically mild bat H18N11 influenza A virus infection^18–21^. Previous work also established the susceptibility of Jamaican fruit bat intestinal organoids to SARS-CoV-2 infection, with increased viral RNA and subgenomic RNA detected in cell lysates and supernatants^22^. Previous work also supports initial susceptibility to Lineage A SARS-CoV-2 infection^23^; Burke et al. challenged Jamaican fruit bats with SARS-CoV-2 Lineage A, which allowed some detection of antigen for two days in the intestinal, but not the respiratory, tract after an inoculation that targeted virus deposition to the trachea and the esophagus. The Delta variant of concern of SARS-CoV-2 demonstrates increased entry fitness in humans^24,25^, as well as transmissibility and pathology. It can, therefore, be hypothesized that this bat species would demonstrate increased susceptibility to this variant intranasal and oral inoculation, which would allow improved untangling of the host response to SARS-CoV-2 in a reservoir host model without the need for physiologically altered or transduced ACE2 overexpression. In this study, we investigated the ability of Delta to infect and replicate in Jamaican fruit bats.

## Results

### Jamaican fruit bat colony

We confirmed the *Artibeus jamaicensis jamaicensis* phylogeny of our bat colony by mtDNA analysis (Supplementary Fig. 1). We then confirmed the health status of the colony. Combined data from 114 uninfected bat transcriptome libraries across 12 tissues generated 2,345,827,700 raw sequence reads. Using the metavirs pipeline^26^, a total of 960,868 reads had matches to viral sequences. Supplementary Table 1 shows a summary of the de novo assemblies for Megahit and Metaspades and Supplementary Fig. 2 displays the Krona chart for the Megahit CAT data. The most abundant virus detected was *Desmodus rotundus* endogenous retrovirus, and no viruses of concern (human or animal pathogens) were detected.

### Jamaican fruit bat ACE2 receptor- and cell-susceptibility

**Fig. 1:**
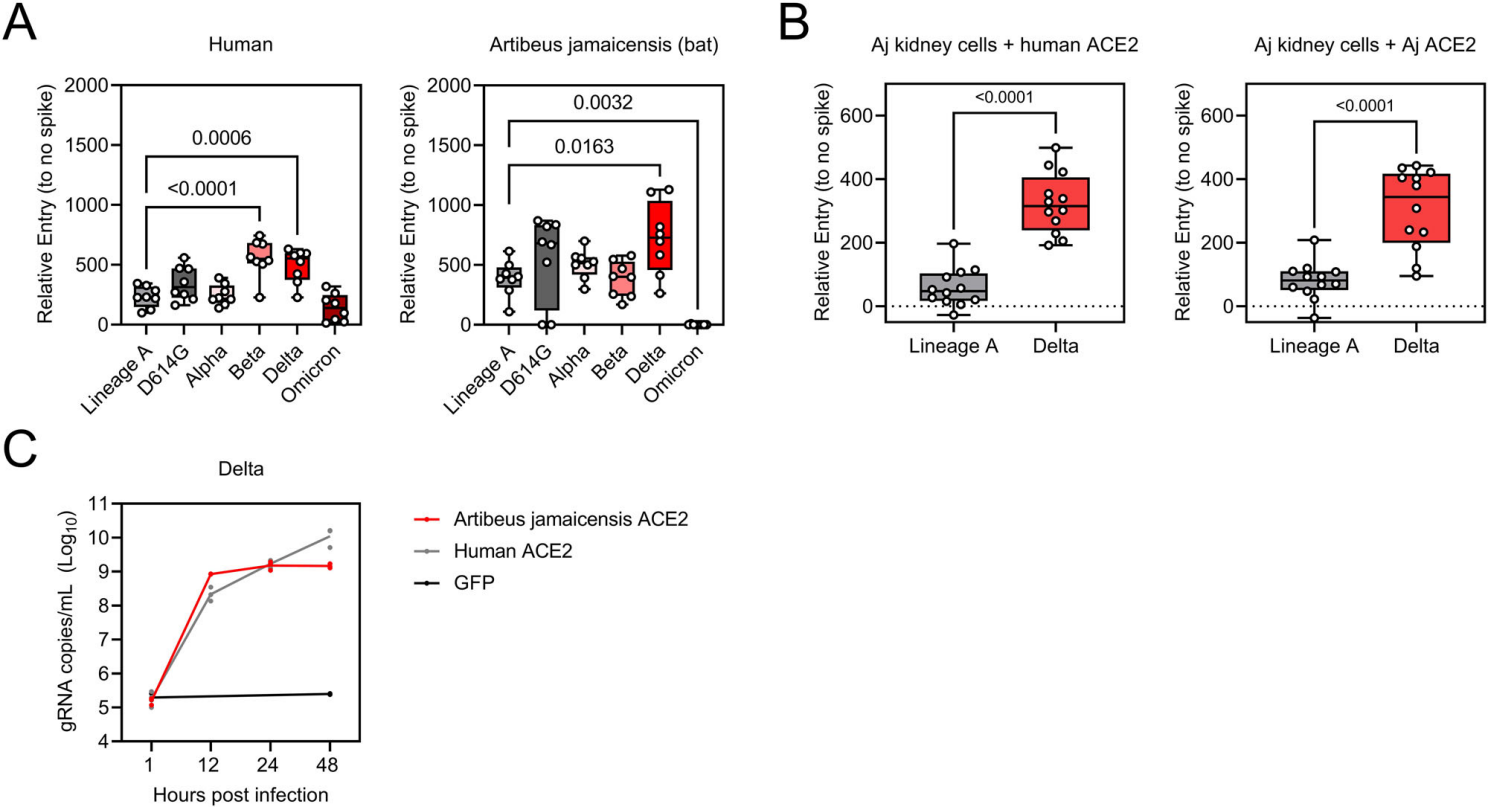
Receptor compatibility of Jamaican fruit bats and SARS-CoV-2 Delta. **A** Relative entry of pseudovirus particles expressing the spike of SARS-CoV-2 variants into human or Jamaican fruit bat ACE2-transfected BHK cells. Relative entry to particles expressing no spike. *N* = 8, ordinary two-way ANOVA, followed by Dunnett’s multiple comparisons test. Whisker-plot depicting min, max, median, and individuals. **B** Relative entry of pseudovirus particles expressing the spike of SARS-CoV-2 variants into human or Jamaican fruit bat ACE2-transfected Jamaican fruit bat immortalized kidney cells. Relative entry to particles expressing no spike. *N* = 12, unpaired *t*-test. Whisker-plot depicting min, max, median, and individuals. **C** Growth kinetics of Delta on human or Jamaican fruit bat ACE2-transfected BHK cells, measured by the presence of gRNA in cell culture supernatant. *N* = 3, multiple unpaired *t*-tests. *p*-Values are indicated where significant.

ACE2, the receptor of SARS-CoV-2, is an important host restriction factor. First, we assessed the capacity of SARS-CoV-2 to enter cells expressing the Jamaican fruit bat (Aj) ACE2. We evaluated the entry of ancestral Lineage A, D614G variant, Alpha, Beta, Delta, and Omicron (BA.1) using a VSV-pseudotype entry assay on human and Aj ACE2 transfected cells^9^. For human ACE2, entry by Beta and Delta was significantly higher than that of Lineage A (mean difference relative to no spike = 326 and 260.5, respectively, *p* < 0.0001 and 0.0007, respectively, *N* = 8, ordinary two-way ANOVA, followed by Dunnett’s multiple comparisons test). For Aj ACE2, entry of Delta was also significantly higher than that of Lineage A (median fold increase relative to no spike = 334.3, *p* = 0.0163), while entry for Omicron was decreased (mean difference increase relative to no spike = −398.9, *p* = 0.0032) (Fig. 1A). For the D614G and Delta variants on the Aj ACE2 transfected cells, increased variability of the data was seen as compared to the other variants and to human ACE2. We proceeded to investigate whether this advantage of Delta was retained in humans and Aj ACE2 transfected Aj immortalized kidney cells. In both human and Aj ACE2 transfected cells, Delta demonstrated significantly increased entry (mean difference relative to no spike = 264.8 or 225.6, respectively, *p* < 0.0001, *N* = 12, unpaired *t*-test) (Fig. 1B). Furthermore, Aj ACE2 supported Delta replication on transfected BHKs; no significant differences were found between the replication efficiency on human or Aj ACE2 transfected cells (multiple unpaired *t* tests) (Fig. 1C). These data suggest that Aj ACE2 facilitates entry of SARS-CoV-2, especially the Delta variant.

### ACE2 and TMPRSSII expression profile in Jamaican fruit bats

**Fig. 2:**
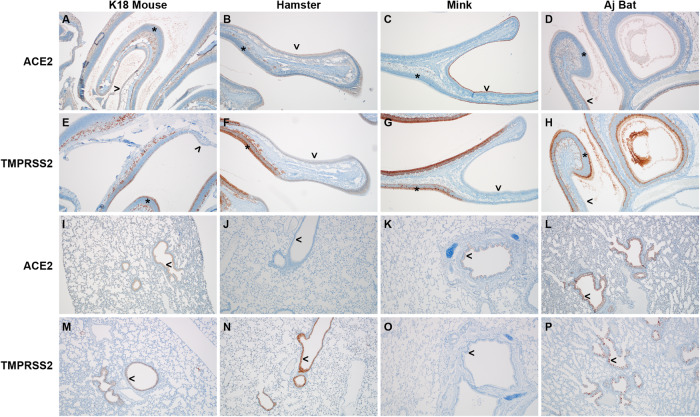
Distribution of ACE2 and TMPRSSII in the upper and lower respiratory tract. Anti-ACE2 immunohistochemistry (**A**–**D**, **I**–**L**) and anti-TMPRSS2 immunohistochemistry (**E**–**H**, **M**–**P**) in k18 humanized mice, Syrian golden hamster, mink, and Jamaican fruit bats. Nasal turbinates: **A** k18 mouse, ACE2 immunoreactivity along the ciliated border of the respiratory epithelium lining the nasal turbinate (>), within rare sustentacular cells of the olfactory epithelium (*) and frequently within olfactory gland cells. There is a non-specific “blush” staining of the submucosa and nasal cavity exudate. **E** k18 mouse, TMPRSS2 immunoreactivity is present in the sustentacular cell layer of the olfactory epithelium (*) and olfactory gland cells but absent in the ciliated respiratory epithelium (>). **B** Hamster, ACE2 minimal apical immunoreactivity of the ciliated respiratory epithelium (>) and a near absence of olfactory epithelial immunoreactivity (*). **F** Hamster, TMPRSS2 immunoreactivity is absent in the ciliated respiratory epithelium (>) but abundant in the sustentacular cell layer of the olfactory epithelium (*) and the glandular epithelium within the submucosa. **C** Mink, ACE2 immunoreactivity of the ciliated respiratory epithelium (>) and absence of olfactory epithelial immunoreactivity (*). **G** Mink, TMPRSS2 immunoreactivity is absent in the ciliated respiratory epithelium (>) but abundant in the sustentacular cell layer of the olfactory epithelium (*) and occasionally the olfactory neuroepithelium. **D** Jamaican fruit (Aj) bat, ACE2 immunoreactivity along the ciliated border of the respiratory epithelium lining the nasal turbinate (>) but absent within the olfactory epithelium (*). **H** Bat, TMPRSS2 immunoreactivity is present in the sustentacular cell layer of the olfactory epithelium (*) and glandular epithelium but absent in the ciliated respiratory epithelium (>). There is also immunoreactivity of the nasal cavity contents, which are believed to be the apical surface of adjacent cells and a result of the function of the cut. Lung: **I**. k18 mouse, ACE2 immunoreactivity spans the apical border of the ciliated and non-ciliated terminal bronchiolar epithelial cells (>). **M** k18 mouse, TMPRSS2 immunoreactivity is both cytoplasmic and nuclear within columnar cells lining the bronchioles (>). **J** Hamster, ACE2 immunoreactivity is absent from the bronchiolar epithelium (>). **N** Hamster, TMPRSS2 immunoreactivity is abundant within the bronchiolar epithelium (>). **K** Mink, ACE2 immunoreactivity of the apical aspect of the bronchiolar ciliated respiratory epithelium (>). **O** Mink, TMPRSS2 immunoreactivity is absent in the bronchiolar ciliated respiratory epithelium (>). **L** Bat, ACE2 immunoreactivity of the bronchiolar ciliated respiratory epithelium (>). **P** Bat, TMPRSS2 immunoreactivity of columnar epithelial cells (>) juxtaposed with mucus-containing goblet cells.

We then compared the expression of both ACE2 and TMPRSII in the upper and lower respiratory tract of Jamaican fruit bats with k18 humanized mice^27^, Syrian Golden hamsters^28^, and mink^29^, highly susceptible animal models for SARS-CoV-2. Distribution of ACE2 and TMPRSS2 remained mostly consistent for the nasal turbinate epithelium and lungs across these four species (Fig. 2). In the upper respiratory tract, we noted ACE2 within the ciliated respiratory epithelium of all four species. In contrast, ACE2 expression was decreased (k18 mice) to absent within the olfactory epithelium. TMPRSS2 immunoreactivity was absent from the respiratory epithelium but present throughout the sustentacular cell layer of the olfactory epithelium and basal cell layer. TMPRSSII was also detected in a portion of the submucosal glandular epithelial cells in k18 mice, hamsters, and bats, but not mink. Within the lungs, ACE2 and TMPRSII were present within the bronchiolar epithelium but not the alveoli across all species. The ciliated respiratory epithelial lining the bronchioles was immunoreactive for ACE2 in k18 mice, mink, and bats, but not hamsters. Conversely, the ciliated respiratory epithelium was immunoreactive for TMPRSSII in k18 mice, hamsters, and bats but not mink.

### Clinical signs in Jamaican fruit bats inoculated with SARS-CoV-2 Delta

**Fig. 3:**
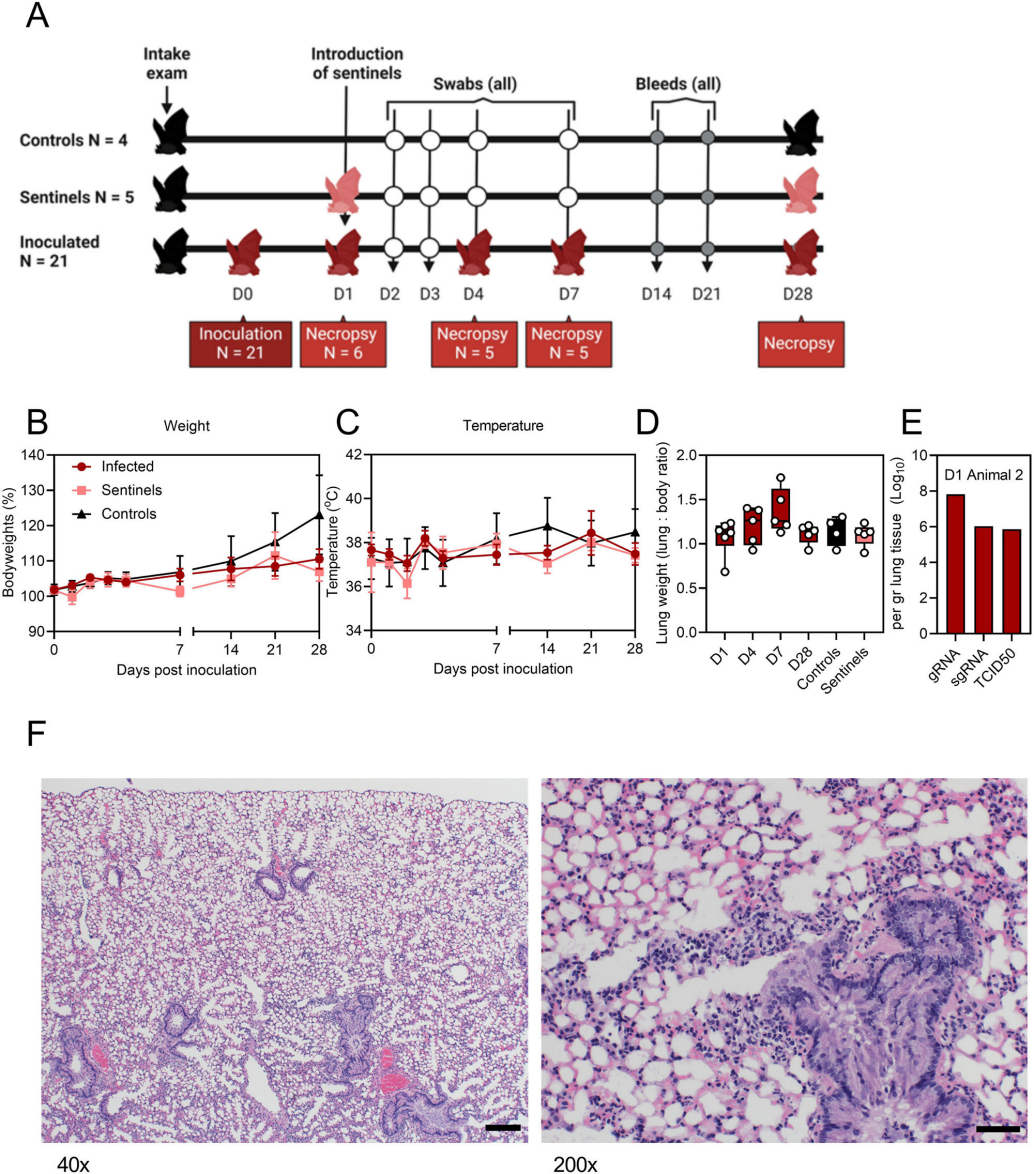
Inoculation of Jamaican fruit bats with SARS-CoV-2 Delta. **A** Schematic representation of the study design. Following an intake exam including pre-bleed, twenty-one bats were inoculated via the intranasal and oral routes with a total of 1.5 × 105 TCID_50_ of SARS-CoV-2 Delta. Four mock-inoculated controls were included. On day 1, 5 sentinels were co-housed with inoculated bats. Six inoculated bats were euthanized on day 1, 5 on day 4, 5 on day 7, and all remaining animals on day 28. Additional blood draws occurred on days 14 and 21. Bats were weighed, temperature was measured, and oropharyngeal and rectal swabs were taken on days 0, 1, 2, 3, 4, 7, 14, 21, and 28. **B** Weight change post-inoculation. Line graph depicting mean and standard error of the mean (SEM). **C** Temperature change post-inoculation. Line graph depicting mean and SEM. **D** Lung weights (measured as lung weight to body weight ratio). Whisker plots depicting minimal and maximal values, median and all individuals. **E** Genomic (g), subgenomic (sg) RNA, and infectious virus titer of the lung samples collected on day 1 from bat 2 Dark red = infected, black = uninfected controls, light red = co-housed sentinels. *p*-Values are indicated where significant. Schematic was generated using BioRender.com. **F** Lung pathology (H&E staining) of a lung collected on day 1. Left 40×, right 200×. Note the increased cellularity around the terminal bronchiole and adjacent alveoli in the 200× photomicrograph.

Twenty-one bats were inoculated via the intranasal and oral routes with a total of 1.5 × 10^5^ TCID_50_ of SARS-CoV-2 Delta. Four mock-inoculated controls were included. On day 1, five sentinel bats were co-housed with inoculated bats (Fig. 3A, Supplementary Table 2). Six inoculated bats were euthanized at 1 DPI, five at 4 DPI, and five at 7 DPI, and the remaining animals, including controls and sentinels, were monitored through 28 DPI. None of the bats showed signs of disease, weight loss (Fig. 2B), or changes in body temperature (Fig. 2C). Sera were collected prior to inoculation and at necropsy. All bats were seronegative for SARS-CoV-2 prior to inoculation. No bat developed a SARS-CoV-2 RBD-specific antibody response by 14, 21, or 28 DPI, measured by ELISA, and no post-challenge serum sample had neutralizing capacity against Delta (Supplementary Table 3).

### Delta replication in Jamaican fruit bats and lung pathology

Tissues collected at the sequential necropsy dates were analyzed for the presence of viral RNA and infectious virus and evaluated by histopathology and immunohistochemistry. We collected lung weights, a sign of SARS-CoV-2 induced pathology, at necropsy. A non-significant trend toward increased lung weights was observed on 4 DPI (Kruskal–Wallis test, followed by Dunn’s multiple comparison test, mean rank difference = 2.25, *p* > 0.9999) and 7 DPI (mean rank difference = 7.250, *p* > 0.9999) (Fig. 3D). However, we were unable to detect RNA or infectious virus in nasal turbinates, bladder, intestinal tract, liver, and kidney (Supplementary Table 5). Viral gRNA (7.8 copies/gr (Log_10_)), sgRNA (6.0 copies/gr (Log_10_)), and infectious virus (5.9 copies/gr (Log_10_)), were detected in 1/6 lungs on 1 DPI in bat 2 (Fig. 3E). SARS-CoV-2-specific immunohistochemistry did not detect the virus in the lung lobes, trachea, nasal turbinates, brain, intestinal tract, liver, spleen, kidneys, bladder, reproductive organs, or heart of any animals. In line with this observation, only minimal pathology was observed, with most bats presenting a complete absence of pathological changes. Radiographs revealed no significant changes at 1 and 4 DPI as compared to 0 DPI (Supplementary Table 4). Only bat 4 demonstrated very minor pathological changes on 4 DPI, whereas all others retained an absence of any pathology (score 0). Minimal interstitial infiltrates were found by histopathological analysis in bats 1 and 2 (2/6) at 1 DPI and in bat 9 at 4 DPI (1/5) (Supplementary Table 4). No abnormal pathology was found in the skull, trachea, brain, intestinal tract, heart, kidney, spleen, and bladder; however, moderate to marked vacuolar change was noted in all livers, including uninfected controls. The cause of this liver change was not identified but may be related to diet or stress. To examine SARS-CoV-2 shedding in inoculated sentinels and control bats, oral and rectal swabs were collected. No viral shedding was observed in any bat at any time point (Supplemental Table 5).

### Intestinal metabolite responses to inoculation with Delta

**Fig. 4:**
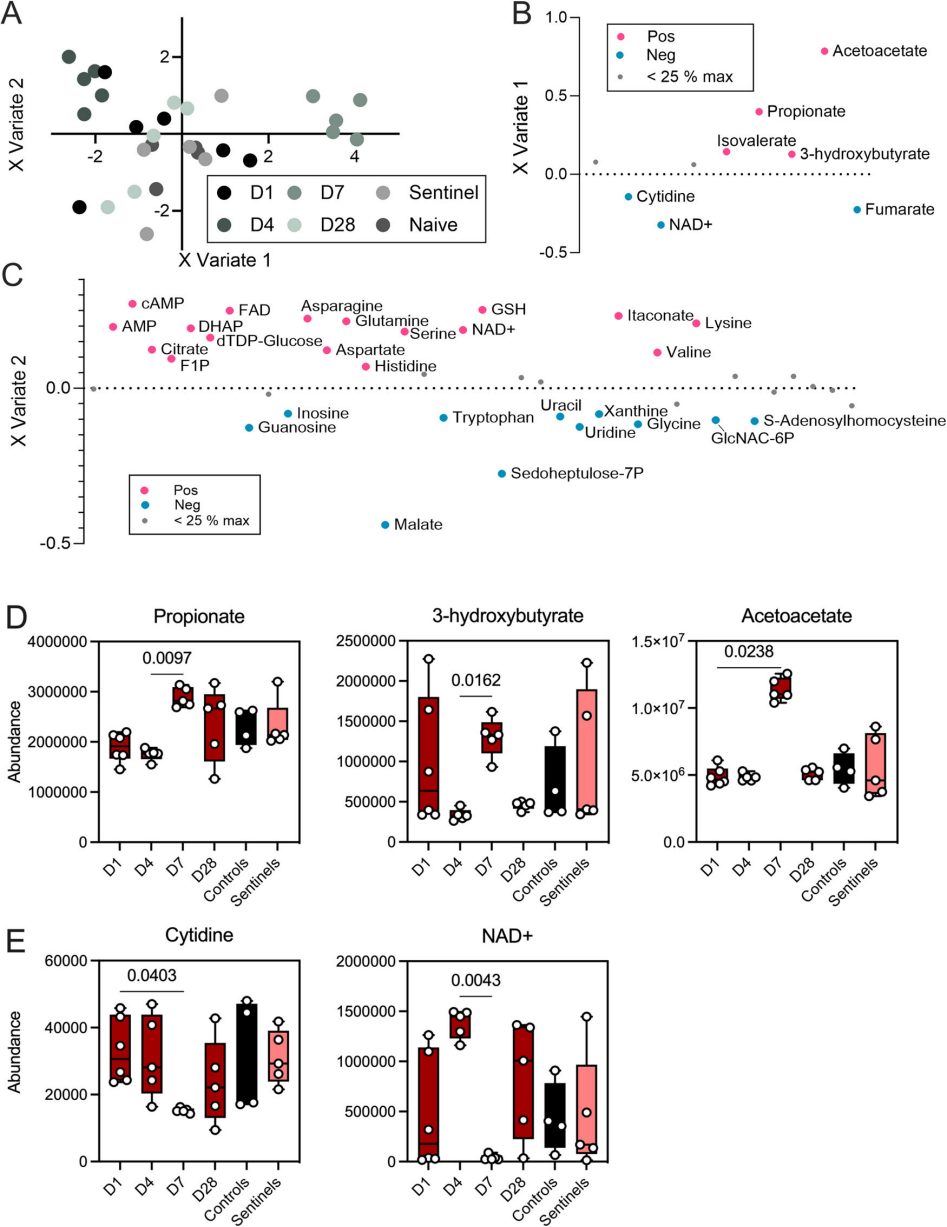
Longitudinal intestinal metabolome changes after inoculation of Jamaican fruit bats with SARS-CoV-2 Delta variant. **A** Sparse partial least squares discriminant analysis (sPLSDA) showing bat intestinal metabolomes by time and inoculation status based on differences in relative metabolite abundance. Points are colored by time and inoculation status. **B** Loadings of *x* variate 1 of sPLSDA analysis of metabolite differences over time. Metabolites greater than or equal to 25% of the maximum absolute loading value are colored according to the direction in which they contribute to variates and labeled with metabolite names. Metabolites less than 25% of the maximum absolute loading value are shown in gray and unlabeled. **C** Loadings of *x* variate 2 of sPLSDA analysis of metabolite differences over time. Metabolites greater than or equal to 25% of the maximum absolute loading value are colored according to the direction in which they contribute to variates and labeled with metabolite names. Metabolites less than 25% of the maximum absolute loading value are shown in gray and unlabeled. **D** Abundances of metabolites that were significantly higher at 7 DPI over the course of the 28-day study period. Median and 95% confidence intervals with individual points overlaid. *P*-values indicated; Kruskal–Wallis test (*N* = 6 (1 DPI)/5 (4, 7, 28 DPI, sentinels)/4 (naïve animals)). **E** Abundances of metabolites that were significantly lower at 7 DPI over the course of the 28-day study period. Median and 95% confidence intervals with individual points overlaid. *p*-Values indicated; Kruskal–Wallis test (*N* = 6 (1 DPI)/5 (4, 7, 28 DPI, sentinels)/4 (naïve animals)).

We analyzed metabolites present in the intestinal tract of each bat at the time of euthanasia (1, 4, 7, or 28 DPI) to determine the effect of inoculation on intestinal metabolite composition. After running a sparse partial least squares-discriminant analysis (sPLSDA), we observed some separation of inoculated bat samples gathered on 4 DPI and pronounced separation of inoculated bat samples gathered on 7 DPI from both inoculated bat samples gathered on 1 or 28 DPI and samples gathered from sentinel and naïve animals (Fig. 4A). 4 DPI samples clustered slightly along *x* variates 1 and 2, and 7 DPI samples clustered strongly along x variate 1. We then visualized the contributions of each metabolite to these two variates, finding that acetoacetate and various SCFAs contributed strongly to *x* variate 1 (Fig. 4B), and thus the 7 DPI metabolomics differences, while a diverse array of metabolites contributed to the 4 DPI differences along *x* variate 2 (Fig. 4C).

When we investigated changes in abundance of the metabolites contributing to x variate 1, we found that five of the seven metabolites were significantly different at 7 DPI as compared to either 1 or 4 DPI (Fig. 4D, E). Propionate and 3-hydroxybutyrate were significantly elevated in 7 DPI samples as compared to 4 DPI samples (*p* = 0.0097 and 0.0162, respectively) and acetoacetate was elevated compared to 1 DPI samples (*p* = 0.0238). Moreover, levels of cytidine were significantly lower in 7 DPI samples than in 1 DPI sample (*p* = 0.0403), while levels of NAD+ were significantly lower in 7 DPI samples than in 4 DPI samples (*p* = 0.0043).

Fitting of the sPLSDA model was assessed with a receiver operating characteristic curve (ROC curve) and the area under the ROC curve (AUROC), with the metabolome being highly predictive of 4 and 7 DPI post-infection with AUROC values of 0.968 and 1, respectively (Supplementary Fig. 3). Taken together, these data indicate significant alterations of some components of the metabolome of bats inoculated with SARS-CoV-2 at 4 and 7 DPI, with a return to baseline by 28 DPI, despite absence of efficient viral replication and signs of disease.

### Transcriptional changes after Delta inoculation

**Fig. 5:**
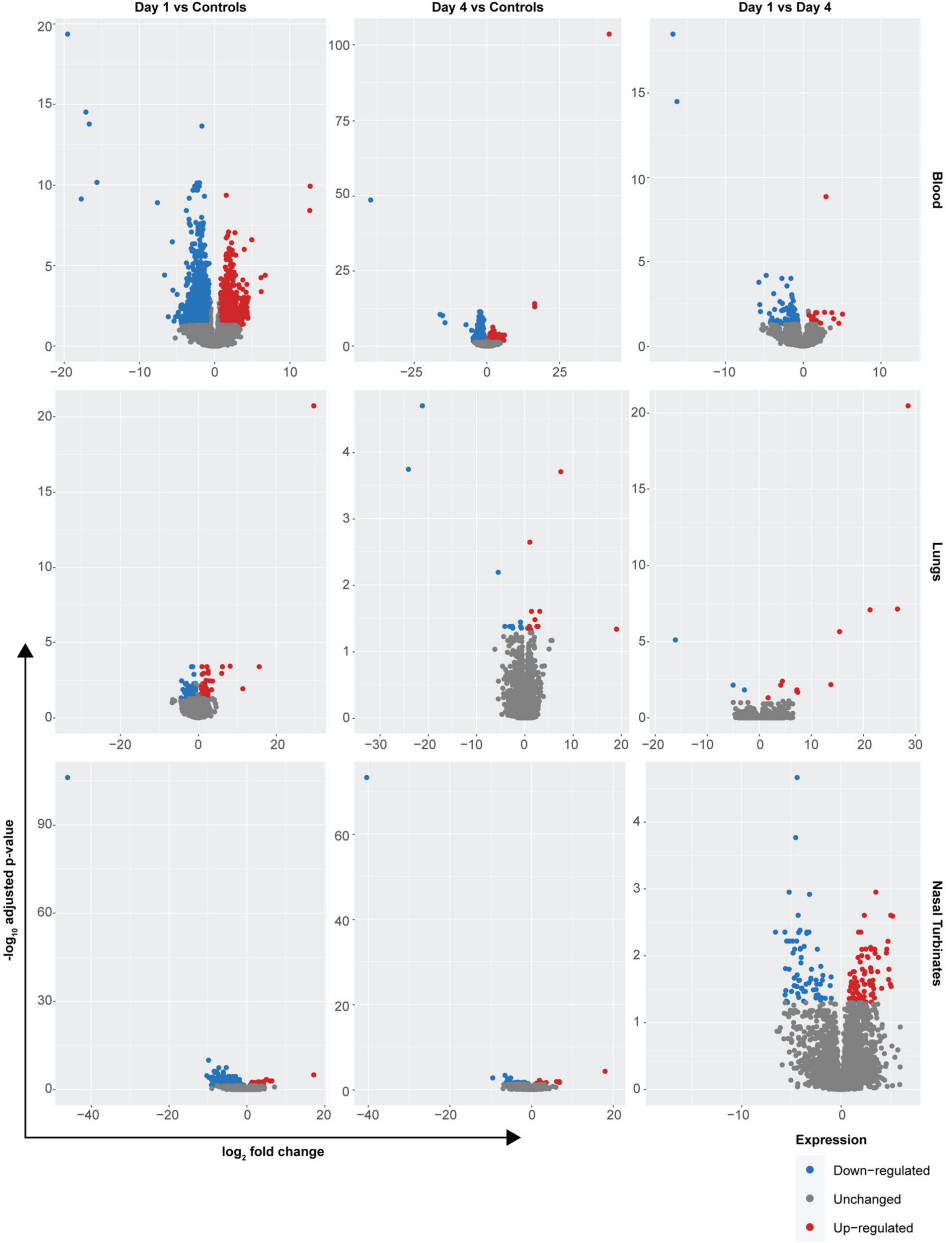
Transcriptional changes in Jamaican fruit bat respiratory tract after inoculation with SARS-CoV-2 Delta. Gene expression changes in blood, lungs, and nasal turbinates collected at 1 and 4 DPI. Tissues collected from uninfected bats at day 28 served as comparison. The expression level of differentially expressed genes (DEGs) was plotted against the significance level in a volcano plot. Shown are DEGs from transcriptomic data collected at 1 and 4 DPI compared to each other or to non-infected animals. Upregulated DEGs are shown in red, downregulated DEGs in blue and non-differentially expressed transcripts are shown in gray.

Even though no robust virus replication was observed, we investigated whether any transcriptional changes in gene expression occurred systemically (blood) and in the lower (lungs) or upper respiratory tract (nasal turbinates), which could provide an explanation for this finding. We specifically investigated the response immediately after inoculation (1 DPI, *N* = 6) and compared it to the response seen at 4 DPI (*N* = 5), where we expected differential upregulation of early innate immune genes. Samples collected from control animals on 28 DPI (*N* = 4) served as gene expression controls under steady-state conditions. No strong difference was observed between groups; most signatures were detected in blood, however read counts were low (Fig. 5, Supplementary Table 6). Comparing 1 DPI with controls, we found 839 upregulated and 1079 downregulated genes in blood, 62 upregulated and 61 downregulated in lungs, and 60 upregulated and 180 downregulated genes in nasal turbinates (significance level = 0.05, foldchange > 2). Using Ingenuity Pathway Analysis, we found that the differential gene expression in blood was associated primarily with significant changes in the canonical integrin signaling and IL-8 signaling pathways (negative *z*-score; −log(*p*-value) > 10) (Supplementary Fig. 3). Even less significant gene up- or downregulations were observed when comparing 4 DPI with controls (Supplementary Tables 6 and 7). Comparing 4 DPI with controls, we found 358 upregulated and 545 downregulated genes in blood, 12 upregulated and 10 downregulated in lungs, and 11 upregulated and 57 downregulated genes in nasal turbinates (significance level = 0.05, foldchange > 2). This was associated primarily with significant changes in the canonical ID1 signaling and the pathogen-induced cytokine storm signaling pathways (negative *z*-score; −log(*p*-value) > 8) (Supplementary Fig. 4).

### Delta infection of Jamaican fruit bat cell lines

**Fig. 6:**
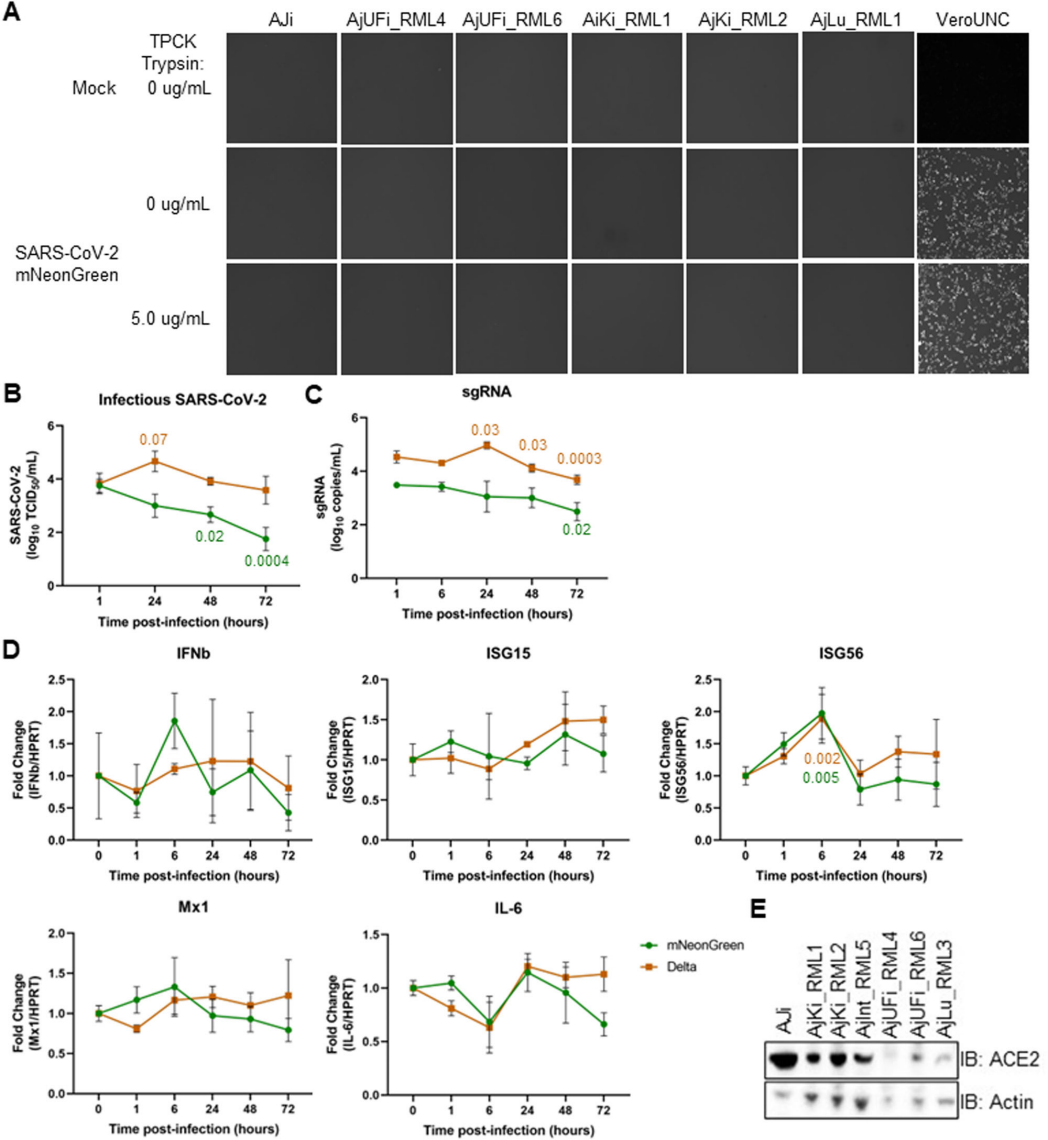
SARS-CoV-2 Delta infects Jamaican fruit bat cells inefficiently. Immortalized kidney (AJi), primary kidney (AjKi_RML1 and 2), primary uropatagium-derived fibroblasts (AjUFi_RML4 and 6), and primary lung (AjLu_RML1) Jamaican fruit bat cells and Vero E6 cells were infected with recombinant SARS-CoV-2 mNeonGreen at multiplicity of infection (MOI) 3.0 in the presence or absence of 5.0 μg/mL TPCK trypsin. **A** Fluorescent images were taken for each condition on duplicate wells 24 h post-infection (representative image shown). **B**–**D** AjKi_RML2 cells were infected with SARS-CoV-2 Delta or mNeonGreen at MOI 3.0. Cellular RNA and supernatant were collected in triplicate to measure intracellular viral infectious virus (**B**), sgRNA (**C**), and host gene expression (**D**). **E** Lysates from immortalized and primary Jamaican fruit bat cells were evaluated for ACE2 protein expression via western blot. One-way ANOVA with Dunnett’s.

Considering the conflicting entry data and in vivo results, we evaluated whether Jamaican fruit bat cells can support SARS-CoV-2 infection and replication in vitro using SARS-CoV-2 mNeonGreen, a recombinant of the Lineage A (WA-1) strain expressing fluorescent reporter NeonGreen. We inoculated immortalized kidney (AJi), primary kidney (AjKi_RML1 and 2), primary uropatagium-derived fibroblasts (AjUFi_RML4 and 6), primary lung (AjLu_RML1), and Vero E6 cells with SARS-CoV-2 mNeonGreen at MOI 3.0. At 24 h post-infection, no fluorescence was observed in any of the Jamaican fruit bat cells, whereas fluorescence was apparent in inoculated Vero cells (Fig. 6A). To test whether spike cleavage presents as a barrier to entry, 5.0 μg/mL TPCK trypsin was added to the virus inoculum and media after infection, but no fluorescence was detected (Fig. 6A).

Because we observed higher entry of SARS-CoV-2 Delta pseudoparticles into cells over-expressing Jamaican fruit bat ACE2, we tested whether SARS-CoV-2 Delta could replicate in actual Jamaican fruit bat cells. We inoculated AJi, AjInt_RML5 (primary intestine), AjKi_RML1, and AjKi_RML2 with MOI 3.0 of either Delta or rSARS-CoV-2 NG for 72 h. Only AjKi_RML2 cells supported minimal production of infectious SARS-CoV-2 Delta at 24 h post-infection (Fig. 6B, Supplementary Fig. 5). A slight increase in sgRNA accompanied virus production (Fig. 6C). No induction of innate antiviral immune responses was detected in either rSARS-CoV-2 NG or Delta infected AjKi_RML2 cells (Fig. 6D). We confirmed that all cell lines tested for SARS-CoV-2 replication express some level of ACE2 protein (Fig. 6E). Interestingly, the kidney cell lines tended to have the highest level of ACE2 while the intestinal and lung cell lines expressed the lowest ACE2 protein. Despite the AJi cells having the highest ACE2, these cells did not support viral replication, suggesting a factor downstream of receptor binding influences the replication phenotype. Overall, these results suggest that Aj kidney cells are minimally susceptible to SARS-CoV-2 Delta, but they are not permissive for infection.

## Discussion

To assess susceptibility to a virus, cell entry and binding analyses are among the first steps taken to demonstrate whether a virus is compatible with the host receptor. In line with this, multiple studies focused on the interaction of the SARS-CoV-2 spike with bat ACE2s, analyzing binding, affinity, and entry^5,7,8^ by using pseudotype or wildtype viruses coupled with in silico predictions to assess the roles of bats in the virus’s origin, the use of different bat species as model systems, or the risk of reverse spillover. Past work with both coronaviruses and viruses of other families has highlighted that in vitro work with cell lines or even organoids is not constitutively indicative of in vivo susceptibility to infection. Work in horseshoe bat intestinal organoids recapitulated the species suspected susceptibility to bat CoV-HKU4^30^. Leschenault’s rousette (*Rousettus leschenaultii)* organoids did not support SARS-CoV-2 infection^31^, whereas in vivo inoculation of Egypian rousettes (*Rousettus aegypticu*s) was successful^13^. WIV1-CoV was unable to cause a robust infection in Egyptian rosettes, even though pseudotype entry confirmed a match between the virus and the bat receptor^12^.

Work in Jamaican fruit bat intestinal organoids suggested that the species would be susceptible to SARS-CoV-2 infection^22^ and could serve as a reliable model to study infection and anti-viral immunity against this virus in this bat. Here, we demonstrate that even though SARS-CoV-2 Delta can use Jamaican fruit bat ACE2 for cell entry to a higher degree than SARS-CoV-2 Lineage A, the species is unable to support robust in vivo infection after a combination of oral and intranasal inoculation; we observed no seroconversion, no shedding, no clinical signs, and infectious virus in only one lung sample on day 1. This contrasts with the findings by Burke et al., where the authors report that the challenge of Jamaican fruit bats with SARS-CoV-2 Lineage A allowed the detection of antigen for 2 days in the intestinal but not the respiratory tract. They also observed no seroconversion and no transmission^23^. Sarbecoviruses are suggested to have an intestinal tract tropism in bat hosts, with virus RNA found in fecal samples and a suggested fecal-oral transmission route^32–34^. Coupled with our findings, this may suggest two things: While the ancestral Lineage A was able to abortedly infect the gastrointestinal tract of Jamaican fruit bats, the more human-adapted Delta variant cannot. It is possible that this is a consequence of adaptation away from the intestinal and towards the respiratory tract in humans after a change in receptor and co-receptor affinity^35^. It is also possible that intragastric inoculation with Delta, as opposed to our oral/nasal inoculation, would have increased the chance of replication in the intestinal tract. However, both hamster and mouse work, as well as epidemiological data in humans, show that the Delta variant infects the gastrointestinal tract of these species^36,37^. SARS-CoV-2 can be considered an enteric pathogen^38^; beyond work in organoids and intestinal cell lines, intranasal, intragastric, and intratracheal inoculation with SARS-CoV-2 led to infection of intestinal segments ranging from the stomach to rectum, suggesting that the virus can survive passaging though the gastric system intact^39^. Higher basal expression of antiviral genes in intestinal tract horseshoe bat organoids curbed coronavirus replication^30^, and SARS-CoV-2 Lineage, A infection of Jamaican fruit bat organoids, led to increased inflammatory signaling, cell turnover, and cell repair^22^. It is feasible that the innate antiviral immune response in these bats against Delta is increased. Additionally, due to differences in ACE2 and protease availability, the mechanism that aborts intestinal tract infection with Lineage A, which the organoid work has highlighted, could be increased in the respiratory tract. It is also possible that intestinal tract antigen was not detected as a result of either a mismatch in sampling times or the collection of the wrong intestinal tract segments.

Metabolomic changes in the intestinal tract can shed light on viral infection kinetics. Our data collected on 4 and 7 DPI post-inoculation does not contradict a potential limited infection of the gut. We observed distinct metabolic events, which may indicate a virally associated process directly in the gut. However, they may also be a consequence of an interaction between the host and virus or non-infectious viral products elsewhere in the body, as the gut is known to respond to infection at distal sites, including the pulmonary compartment^40^. The day 4 metabolic event is dominated by the elevation of NAD+ and other redox factors, including FAD+. NAD+ is known to contribute to the regulation of the inflammatory processes in the gut, and decreases in NAD+ in the feces or urine can be associated with increased PARP activity due to stress events and cellular damage at distal sites^41^. Elevated NAD+ may indicate an immune or cellular stress event that preceded and is recovering by the day 4 timepoint. Elevation of the cellular signaling metabolite cAMP on day 4 may also point to recovery from a preceding or still active immune or stress event. The day 7 metabolic event is characterized by elevated SCFAs, including propionate and isovalerate. SCFAs are well-established markers of gut health and suppression of inflammation^42^. Propionate is known to affect components of both the adaptive and innate immune system as well as exert a direct effect on microflora in other species^43^. Thus this SCFA event may represent further immunomodulatory recovery of homeostasis after a subtle immune event within the gut. Whether this is the same event or a subsequent event to the one indicated by the day 4 metabolic signature will require further investigation. These metabolic events could have also been caused by other temporal stresses unrelated to the viral infection so further investigation is necessary to distill the mechanistic role of these metabolic phenotypes. Regardless, both of these metabolic events are resolved completely by day 28 post-infection, confirming that any potential dysbiosis is brief in duration.

We observe strain-specific differences between SARS-CoV-2 Lineage A and Delta on the bat cell lines tested. The lack of fluorescence following SARS-CoV-2 mNeon Green (WA-1, Lineage A) infection indicates no viral transcription occurred. In contrast, sgRNA and minimal amounts of infectious virus are detected after exposure to Delta, confirming the increased entry observed with the pseudotypes. For both variants, treatment with trypsin did not impact the infection phenotype, suggesting a factor other than spike cleavage efficiency blocks replication in Jamaican fruit bats. Although SARS-CoV-2 Delta enters Jamaican fruit bat cells and can replicate its genome, the limited production of infectious progeny virus suggests the presence of a restriction factor, limiting the ability of the virus to replicate in vivo. Despite the presence of intracellular sgRNA in one of the tested cell lines, indicative of a replicating virus, we did not observe increased expression of innate immune genes at 48 h post-infection. The lack of innate immune induction could suggest that the virus is antagonizing the innate immune genes or that the virus is replicating at levels below the threshold required to stimulate a response. However, this hypothesis requires further testing, as observations in one limited permissive cell line cannot completely inform in vivo conclusions. Similarly, however, we did not observe significant upregulation of host anti-viral genes after in vivo infection. Interestingly, even the addition of human ACE2 into the bats through intranasal adenovirus vector infection by Burke et al. only nominally overcame this restriction: though this led to an increased number of ACE2-expressing cells in the lungs, viral RNA could only be found in one out of four bats on days 4 and 7^23^. This suggests that the intrinsic ability of Jamaican fruit bats to restrict replication after entry may, in principle, be overcome through a dose effect, though not efficiently. Additional intestinal tract work in in vitro systems may further shed light on the nature of this restriction. It could have been expected that pseudotype entry and replication in Aj ACE2 transfected cells would predict in vivo susceptibility. However, our study clearly demonstrates the limitations inherent to this approach. To more accurately predict species susceptibility, future infection studies should focus on wild-type virus replication and growth in primary cell lines derived from multiple potential target organs.

Determining the potential host range of novel viruses is beneficial for identifying which species may serve as natural hosts or be at risk of infection. Many studies rely on entry assays to predict host distribution without following up with in vitro or in vivo studies. The host factors that best inform host–virus compatibility will vary, and the development of high-throughput assays to identify essential and restriction factors within the host would enhance our ability to predict host suitability.

## Methods

### Ethics statement

All animal experiments were conducted in an AAALAC International-accredited facility, approved by the Rocky Mountain Laboratories Institutional Care and Use Committee (Protocol number 2021-037-E), and adhered to the guidelines put forth in the Guide for the Care and Use of Laboratory Animals 8th edition, the Animal Welfare Act, United States Department of Agriculture and the United States Public Health Service Policy on the Humane Care and Use of Laboratory Animals. Work with infectious SARS-CoV-2 virus strains under BSL3 conditions was approved by the Institutional Biosafety Committee (IBC) and conducted in BSL-4 facilities. For the removal of specimens from high containment areas virus inactivation of all samples was performed according to IBC-approved standard operating procedures^44^.

### Viruses

Delta variant (B.1.617.2) hCoV19/USA/MD/HP05647/2021, EPI_ISL_2331496, was kindly provided by Andrew Pekosz (John Hopkins Bloomberg School of Public Health, MD, USA). Delta variant hCoV-19/USA/KY-CDC-2-4242084/2021, EPI_ISL_1823618, was obtained from BEI resources. SARS-CoV-2 WA1 (Lineage A) strain expressing Neon Green (SARS-CoV-2 mNeonGreen) was kindly provided to us through Pei-Yong Shi (Department of Biochemistry and Molecular Biology, University of Texas Medical Branch, Galveston, TX, USA)^45^.

Virus propagation was performed in Vero E6 cells (ATCC CRL-1586) in DMEM supplemented with 2% fetal bovine serum (FBS), 1 mM l-glutamine, 50 U/mL penicillin, and 50 μg/mL streptomycin (DMEM2). Vero E6 cells were maintained in DMEM supplemented with 10% fetal bovine serum, 1 mM l-glutamine, 50 U/mL penicillin, and 50 μg/mL streptomycin. No mycoplasma and no contaminants were detected. All virus stocks were sequenced; no SNPs compared to the patient sample sequence were detected in the original Delta stocks EPI_ISL_1823618 and EPI_ISL_2331496. Stock EPI_ISL_1823618 was used for the growth curve infection on transfected baby hamster kidney cells, and EPI_ISL_2331496 for the in vivo infection to obtain a high enough inoculum titer. A new stock EPI_ISL_2331496 was required for the in vitro infection experiments on primary cell lines: One SNP, nsp14 N410N (wobble), was found compared to the patient sample.

### Generation of primary and immortalized cell lines

At necropsy, a sample of uropatagium, lung, kidney, and small intestine was collected in media (10% FBS DMEM/Ham’s F-12 with primocin, penicillin, and streptomycin, amphotericin B, and non-essential amino acids) and placed on ice. Within 4 h of collection, the lung, kidney, and small intestine were cut into smaller sections and digested in media with Liberase TM (Roche, REF 05401127001) while rotating for 1 h at room temperature. The digested tissue was centrifuged at 500*g* for 5 min and then transferred to a T25 flask with fresh media. Within 24 h of collection, a 3 mm section of uropatagium was punch biopsied and washed twice in 1× DPBS. This 3 mm section was cut into smaller sections using a sterile blade and transferred to fresh media with Liberase TM. After incubation at 37 °C with 5% CO_2_ and rotating overnight, the digested uropatagium was centrifuged at 500*g* for 5 min and plated in a T25 flask with 6 mL of fresh media. All cells were confirmed to be mycoplasma negative. Immortalized Jamaican fruit bat kidney cells (AJi) were previously described^9,46^.

### Jamaican fruit bat primary cells in vitro infections

Immortalized (AJi) and primary Jamaican fruit bat cells and Vero E6 cells were plated (250,000 cells/mL, 350 μL) in 48-well plates and incubated at 37 °C with 5% CO_2_. Sixteen hours after plating, media was removed, and cells were inoculated with recombinant SARS-CoV-2 mNeonGreen^45^ in the presence or absence of 5.0 μg/mL TPCK trypsin. One hour after inoculation, cells were washed three times with 1× DBPS and given fresh media with or without 5.0 μg/mL TPCK trypsin. The SARS-CoV-2 mNeonGreen infected cells were monitored for fluorescence using the FITC channel of an ECHO Revolve fluorescence microscope. Supernatants were collected from SARS-CoV-2 Delta and -mNeonGreen inoculated AJi, AjKi_RML1, AjKi_RML2, and AjInt_RML5 cells through 72 h after infection. Cell lysates from infected AjKi_RML2 cells were collected in RLT (QIAGEN) for analysis of viral sgRNA and host gene expression. Supernatants were titrated on Vero-TMPRSII cells. RLT lysates were transferred to ethanol prior to the extraction of RNA using the RNEasy extraction kit (Qiagen). Cellular RNA was used to measure intracellular subgenomic (sg)RNA and host genes using qRT-PCR (Table 1). Fold change in gene expression is presented as (ΔΔC_T_ target gene/ΔΔC_T_ HPRT) of the sample/(ΔΔC_T_ target gene/ΔΔC_T_ HPRT) of the mock average.

**Table 1:** qRT PCR primers for Jamaican fruit bat immune genes

AJ Interferon-beta F	ACTTCAAGTTTCCCGAGGAGA
AJ Interferon-beta P	FAM-GCACGGGCTGGAATGAGACCATCATTGA
AJ Interferon-beta R	GGTCCATCTGCCAACTGAGT
AJ Interferon-stimulated gene 15 F	CAGAAGGTGGCTGAGCTGAA
AJ Interferon-stimulated gene 15 P	FAM-TGGCTGAGTTTCCAGGGGAGGCCC
AJ Interferon-stimulated gene 15 R	CTTGTATTCCTTCAGCTGCGC
AJ Interferon-stimulated gene 56 F	AGAGCTTGAAGCAGGCTGAA
AJ Interferon-stimulated gene 56 P	FAM-ACATGCTGGCCAGTCGGAGGTGAG
AJ Interferon-stimulated gene 56 R	CTTCTAGTCTGCCCATGCGG
AJ MX Dynamin Like GTPase 1 F	GTTCTTCATGCTCCGGTCGT
AJ MX Dynamin Like GTPase 1 P	FAM-GCCAGAAGCTGAGCAATGCCATGTTGC
AJ MX Dynamin Like GTPase 1 R	TTCCTCTTGTCGCTGGTGTC
AJ Interleukin-6 F	AACAGCAAGGAGGCACTGAC
AJ Interleukin-6 P	FAM-ACCTGAACCTTCCGAAACTGACAAGAAG
AJ Interleukin-6 R	CAGACCGGTGGTGAGTCTC
AJ hypoxanthine phosphoribosyltransferase 1 F	AGATGGTGAAGGTCGCAAG
AJ hypoxanthine phosphoribosyltransferase 1 P	FAM-ACTTTGTTGGATTTGAAATTCCAGACAAGTTTG
AJ hypoxanthine phosphoribosyltransferase 1 R	CCTGAAGTATTCATTATAGTCAAGGG

### Western blots

Cell lysates collected in the SDS buffer were run on 4–12% Bis-Tris NuPAGE gels (Invitrogen) at 150 V for 1 h, then transferred onto a methanol-activated PVDF membrane (BioRad). Following a 1-h block in 5% powdered milk, the membranes were washed in wash buffer (1× tris-HCL with 0.1% tween 20) three times. Membranes were probed with primary antibody overnight, rocking at 4 °C. The next day, blots were washed three times, incubated with an HRP-conjugated secondary antibody for 1 h rocking at room temperature, and washed three times before developing. Blots were incubated with a 1:1 ratio of peroxidase and enhancer reagents Clarity Western ECL (BioRad) or SuperSignal West Femto (Thermo Scientific) and developed on an iBright imaging system (Thermo Fisher Scientific). Primary antibodies used: ACE2 (R&D Systems, AP933-SP) and actin (Abcam, ab8227). Secondary antibodies used: Rabbit-anti-goat (Sigma-Aldrich, A5420) and sheep-anti-mouse (GE Healthcare, NA931).

### Entry essays

The spike encoding sequences for SARS-CoV-2 variants Lineage A, D614G, Alpha, Beta, Delta, and Omicron were truncated by deleting 19 aa at the C-terminus. The S proteins with the 19 aa deletions were previously reported to increase the efficiency of incorporation into virions of VSV pseudotypes, which include a firefly luciferase reported inserted into the vector via *MluI* and *AvrII*^47,48^. These sequences were codon optimized for human cells, then appended with a 5’ Kozak consensus sequence (GCCACC) and 3’ tetra-glycine linker followed by nucleotides encoding a FLAG-tag sequence (DYKDDDDK). The spike sequences were synthesized and cloned into pcDNA3.1^+^ (GenScript). Pseudotype production was carried out as described previously^9^. Briefly, plates pre-coated with poly-l-lysine (MilliporeSigma) were seeded with 293 T cells and transfected the following day with 1200 ng of empty plasmid and 400 ng of plasmid encoding coronavirus spike or no-spike plasmid control (green fluorescent protein (GFP)). After 24 h, transfected cells were incubated with VSVΔG seed particles pseudotyped with VSV-G as previously described^9,49^. After 1 h of incubation with intermittent shaking at 37 °C, cells were washed four times and incubated with 2 mL DMEM supplemented with 2% FBS, penicillin/streptomycin, and l-glutamine for 48 h. Supernatants were collected, centrifuged at 500*g* for 5 min, aliquoted, and stored at −80 °C.

Human and Jamaican fruit bat angiotensin-converting enzyme 2 (ACE2) (Q9BYF1.2 and XM_037157556.1, respectively) were synthesized and cloned into pcDNA3.1^+^ (GenScript). All DNA constructs were verified by Sanger sequencing (ACGT). Baby hamster kidney (BHK) cells and immortalized Jamaican fruit bat kidney cells were seeded in black 96-well plates and transfected the following day with 100 ng plasmid DNA encoding human or bat ACE2 using either polyethylenimine (Polysciences) for the BHK cells, or Lipofectamine 3000 (ThermoFisher) for the Jamaican fruit bat kidney cells. All downstream experiments were performed 24 h post-transfection. Transfected cells were inoculated with equivalent volumes of pseudotype stocks. Plates were then centrifuged at 1200*g* at 4 °C for 1 h and incubated overnight at 37 °C. 18–20 h post-infection, Bright-Glo luciferase reagent (Promega) was added 1:1 to each well, and luciferase was measured. Relative entry was calculated by normalizing the relative light unit for spike pseudotypes to the plate relative light unit average for the no-spike control.

### Growth kinetics on transfected BHK cells

BHK cells were seeded for confluency in 24-well plates and transfected the next day with 100 ng plasmid DNA encoding human or hamster ACE2 or GFP, using polyethylenimine (Polysciences). All downstream experiments were performed 24 h post-transfection. Cells were inoculated with Delta (MOI = 0.01) in DMEM. After 1 h, wells were washed twice with 1 mL DMEM (T0), then incubated in 1 mL DMEM supplemented with 2% FBS. Supernatants were collected at T0, T12, T24, and T48 from unique individual wells.

### Bat inoculation

Jamaican fruit bats *(Artibeus jamaicensis)* from a closed colony were utilized for these studies. Animals were cohoused in same-sex groups of up to five animals per cage. The study comprised 30 animals total, 9 males and 21 females, of which 10 were pregnant. Animals were allowed to acclimate to the facility for 5 days. Twenty-one animals were intranasally and orally inoculated with a total of 1.5 ×105 TCID_50_ of hCoV19/USA/MD/HP05647/2021 (B.1.617.2/Delta, EPI_ISL_2331496), and four animals were inoculated with DMEM as controls via the same routes. On day 1, five animals were cohoused as sentinels (Fig. 2A). Caging scheme is depicted in Supplementary Table 2. All inoculations and subsequent manipulations were performed under isoflurane anesthesia. Six SARS-CoV-2 inoculated bats were euthanized 1-day post-inoculation (DPI), and five bats were euthanized on days 4 and 7 post-inoculation to assess viral replication in tissue samples. Fourteen inoculated animals, 5 sentinels, and 4 controls were euthanized 28 days post-inoculation for disease course assessment and shedding analysis. Blood was collected at baseline and on 14 and 21 DPI. For survival blood collections, animals were anesthetized with isoflurane, and a 25 G needle was utilized to puncture the cephalic vein. Blood was then collected into SAFE-T-FILL Capillary collection tubes. Once the required blood volume was collected, manual pressure was applied to the puncture site to ensure hemostasis. At euthanasia, animals were anesthetized with isoflurane, and blood was collected by cardiac puncture with a 23 G needle and syringe. Body temperature was obtained via implanted transponder (BMDS IPTT-300): Sterile, single-use Temperature Transponders/microchips (BMDS IPTT-300, Avidity Science) were programmed with the animal’s identification number and injected subcutaneously along the lateral flank in an anesthetized animal. To obtain the animal’s subcutaneous body temperature, a reader was passed over the animal/implanted transponder, and the animal’s identification number and body temperature were displayed on the reader’s screen. Bats were weighed, and oropharyngeal and rectal swabs were taken on days 0, 1, 2, 3, 4, 7, 14, 21, and 28. Swabs were collected in 1 mL DMEM with 200 U/mL penicillin and 200 µg/mL streptomycin. For the control group, mock swabs were performed to ensure animals underwent the same anesthesia protocols as infection groups and to ensure no accidental contamination occurred between groups. Bats were observed daily for clinical signs of disease. Necropsies and tissue sampling were performed according to IBC-approved protocols. Ventrodorsal, right, and left lateral thoracic radiographs were taken on day 0 and at euthanasia prior to any other procedures. Radiographs were evaluated and scored for the presence of pulmonary infiltrates by two board-certified clinical veterinarians according to a standard scoring system^50^. Briefly, each lung lobe (upper left, middle left, lower left, upper right, middle right, lower right) was scored individually based on the following criteria: 0 = normal examination; 1 = mild interstitial pulmonary infiltrates; 2 = moderate interstitial pulmonary infiltrates, perhaps with partial cardiac border effacement and small areas of pulmonary consolidation (alveolar patterns and air bronchograms); and 3 = pulmonary consolidation as the primary lung pathology, seen as a progression from grade 2 lung pathology. Thoracic radiograph findings were reported as a single radiograph score for each animal. To obtain this score, the scores assigned to each of the six lung lobes were added together and recorded as the radiograph score for each animal. Scores, therefore, may range from 0 to 18.

### Viral RNA detection

Swabs were collected as described above. 140 µL was used for RNA extraction using the QIAamp Viral RNA Kit (Qiagen) using QIAcube HT automated system (Qiagen) according to the manufacturer’s instructions with an elution volume of 150 µL. For tissues, RNA was isolated using the RNeasy Mini kit (Qiagen) according to the manufacturer’s instructions and eluted in 60 µL. Sub-genomic (sg) and genomic (g) viral RNA were detected by qRT-PCR^51^. RNA was tested with TaqMan™ Fast Virus One-Step Master Mix (Applied Biosystems) using QuantStudio 6 or 3 Flex Real-Time PCR System (Applied Biosystems). SARS-CoV-2 standards with known copy numbers were used to construct a standard curve and calculate copy numbers/mL or copy numbers/g.

### Virus titration

Viable virus in tissue samples was determined as previously described^15^. In brief, lung tissue samples were weighed and then homogenized in 1 mL of DMEM (2% FBS). Swabs were used undiluted. Vero E6 cells were inoculated with ten-fold serial dilutions of homogenate, incubated for 1 h at 37 °C, and the first two dilutions were washed twice with 2% DMEM. For swab samples, cells were inoculated with ten-fold serial dilutions and no wash was performed. After 6 days, cells were scored for cytopathic effect. TCID_50_/mL was calculated by the Spearman–Karber method.

### Serology

Maxisorp plates (Nunc) were coated with 50 ng receptor binding domain (RBD) protein (generated and kindly provided by Florian Krammer) per well. Plates were incubated overnight at 4 °C. Plates were blocked with casein in phosphate-buffered saline (PBS) (ThermoFisher) for 1 h at room temperature. Serum was diluted twofold in blocking buffer, and samples (duplicate) were incubated for 1 h at room temperature. Secondary horseradish peroxidase (HRP)-conjugated recombinant A/G protein (Invitrogen, lot number WH 324034) diluted 1:10,000 was used for detection and visualized with KPL TMB two-component peroxidase substrate kit (SeraCare, 5120-0047). The reaction was stopped with KPL stop solution (SeraCare), and plates were read at 450 nm. Plates were washed 3× with PBS-T (0.1% Tween) in between steps. The threshold for positivity was calculated as the mean plus 3× the standard deviation of negative control bat sera.

Virus neutralization. Heat-inactivated, irradiated sera were twofold serially diluted in DMEM, and 100 TCID_50_ of SARS-CoV-2 was added. After 1 h of incubation at 37 °C and 5% CO_2_, the virus:serum mixture was added to Vero E6 cells. CPE was scored after 5 days at 37 °C and 5% CO_2_.

### Histopathology

Necropsies and tissue sampling were performed according to IBC-approved protocols. Tissues were fixed for a minimum of 7 days in 10% neutral buffered formalin with 2 changes. Tissues were placed in cassettes and processed with a Sakura VIP-6 Tissue Tek on a 12-h automated schedule using a graded series of ethanol, xylene, and PureAffin. Prior to staining, embedded tissues were sectioned at 5 µm and dried overnight at 42 °C. Using GenScript U864YFA140-4/CB2093 NP-1 (1:1000), specific anti-CoV immunoreactivity was detected using the Vector Laboratories ImPress VR horse anti-rabbit IgG polymer (# MP-6401) as the secondary antibody. ACE2 was detected using a primary antibody from R&D Systems (cat#AF933, 1:100 dilution) and a secondary anti-goat IgG polymer (ImmPress, Vector Laboratories cat#MP-7405). TMPRSSII was detected using a primary antibody from Abcam (cat#ab109131, 1:2000 dilution) and a secondary horse anti-rabbit IgG polymer (ImmPress VR, Vector Laboratories cat#MP-6401).

The tissues were stained using the Discovery Ultra automated stainer (Ventana Medical Systems) with a ChromoMap DAB kit Roche Tissue Diagnostics (#760-159).

### Next-generation sequencing of mRNA

Tissue sections were collected directly into 1 mL of Trizol (Thermofisher Scientific), 200 µL of 1-bromo-3-chloropropane (MilliporeSigma) was added, samples mixed, and centrifuged at 16,000*g* for 15 min at 4 °C. 600 µL of RNA in the aqueous phase was collected from each sample and passed through Qiashredder column (Qiagen) at 21,000*g* for 2 min to homogenize any remaining genomic DNA, then combined with 600 µL of RLT lysis buffer (Qiagen, Valencia, CA) with 1% beta-mercaptoethanol (MilliporeSigma), and RNA extracted using Qiagen AllPrep DNA/RNA 96-well system. An additional on-column DNase-1 treatment was performed during RNA extraction. RNA was quantitated by spectrophotometry, and yield ranged from 0.4 to 17.8 µg. Two hundred nanograms of RNA were used as input for polyA pull-out and NGS library preparation following the Illumina Stranded mRNA prep workflow (Illumina). The NGS libraries were prepared, amplified for 15 cycles, AMPureXP bead (Beckman Coulter) purified, assessed on a TapeStation 4200 (Agilent Technologies), and quantified using the Kapa Library Quantification Kit (Illumina). Amplified libraries were normalized, pooled at equal 2 nM amounts, and sequenced as 2× 75 bp reads on the NextSeq instrument using three high output chemistry kits (Illumina). Raw fastq reads were trimmed of Illumina adapter sequences using cutadapt version 1.12, and then trimmed and filtered for quality using the FASTX-Toolkit (Hannon Lab). The remaining reads were aligned to the *Artibeus jamaicensis* genome assembly version 1.0 using Hisat2^52^. Reads mapping to genes were counted using htseq-count^53^. Differential expression analysis was performed using the Bioconductor package DESeq2^54^, and data was further analyzed and plotted using ggplot2 (V3.4.0) as part of the tidyverse package (V1.3.2)^55^. Pathway analysis was performed using Ingenuity Pathway Analysis (Qiagen), and gene clustering was performed using Partek Genomics Suite (Partek Inc.).

### Phylogenetic tree

To map the RML colony to the most likely Jamaican fruit bat subspecific lineage, we obtained the entire cytochrome b data set for Jamaican fruit bat from Larsen et al. (2007)^56^, comprising 176 individuals across all documented subspecies (Simmons 2005), as well as additional *Artibeus* species and brown fruit-eating bat (*Koopmania concolor*) to root the phylogeny. In addition, we extracted the cytochrome b locus from the WHU_Ajam2 assembly (https://www.ncbi.nlm.nih.gov/assembly/GCA_014825515.1) as well as the more recently released CSHL_Jam assembly (http://compgen.cshl.edu/bat/). All cytochrome b sequences were then aligned using MAFFT v7.475^57^, and a maximum likelihood phylogeny was generated using RaxML-NG v1.1.0^58^ using the GTR + G substitution model and 1,000 bootstrap replicates; all other parameters were left in default settings. The final phylogeny is rooted in the brown fruit-eating bat for visualization.

### Viral metagenome analysis on uninfected Jamaican fruit bat colony

Per-cycle base call (BCL) files were converted to fastq files and demultiplexed using bcl2fastq v2.20.0.244 (Illumina, Inc. San Diego, CA). Raw fastq files were concatenated for 114 samples across twelve tissues (spleen, brain, liver, upper and lower gastrointestinal tract, Peyer’s patches, blood, kidney, lung, mediastinal and mesenteric lymph nodes, and nasal turbinates) from five female and five male bats, and then processed through Metavirs pipeline^26^ built-in snakemake^59^. Briefly, adapter sequences were trimmed using cutadapt^60^, and reads were filtered for host genomes using Bowtie2^61^. The remaining reads were assembled in parallel using Megahit^62^ and Metaspades^63^, and contigs were annotated using the Contig Annotation Tool^64^ and Kraken2^65^. Results were visualized using Krona^66^.

### Metabolome analysis

A 1 cm sample of ileal tissue was collected from each bat for metabolome analysis at the time of necropsy. Liquid chromatography-mass spectrometry (LCMS) grade solvents were used for all LCMS methods. Tissue samples were immersed directly in 0.4 mL of methanol and shredded at 30 Hz for 10 min using a tissue shredder and one stainless steel bead (Qiagen, 5 mm) per sample. The supernatant was then irradiated at 2 mRad for sample removal from high containment. 0.4 mL of water and 0.4 mL of chloroform were then added to each sample. Samples were shaken for 30 min at 4 °C and centrifuged at 16,000×*g* for 20 min to establish layering. 400 µL of the top (aqueous) layer was collected. The aqueous layer was diluted 5× in 50% methanol in water for LCMS injection. A subaliquot of the aqueous layer was taken for O-benzylhydroxylamine derivatization of carboxylic acids and SCFA analysis.

Samples were derivatized with O-benzylhydroxylamine (O-BHA) according to previously established protocols with modifications^67,68^. A reaction buffer consisting of 1 M pyridine and 0.5 M hydrochloric acid in water was prepared fresh. A volume of 35 µL of the aqueous metabolite extract was sub-aliquoted. 10 µL of 1 M O-BHA in reaction buffer and 10 µL of 1 M 1-ethyl-3-(3-dimethylaminopropyl)carbodiimide in reaction buffer were added to the sample. Samples were shaken at room temperature for 2 h. The reaction was quenched with 50 µL of 0.1% formic acid for 10 min. Derivatized carboxylic acid compounds were extracted via the addition of 400 µL ethyl acetate. Following mixing and centrifugation at 16,000×*g* for 5 min at 4 °C to induce layering, the upper (organic) layer was collected and dried under vacuum. Samples were resuspended in 300 µL of water for LCMS injection.

Tributylamine and all synthetic molecular references were purchased from Millipore-Sigma. LCMS-grade water, methanol, isopropanol, and acetic acid were purchased through Fisher Scientific. All samples were separated using a Sciex ExionLC™ AC system and measured using a Sciex 5500 QTRAP® mass spectrometer. Aqueous metabolites were analyzed using a previously established ion pairing method with modification^69^. Quality control samples were injected after every 10 injections. Samples were separated on a Waters™ Atlantis T3 column (100 Å, 3 µm, 3 mm × 100 mm) and eluted using a binary gradient from 5 mM tributylamine, 5 mM acetic acid in 2% isopropanol, 5% methanol, 93% water (v/v) to 100% isopropanol over 15 min. Two distinct MRM pairs in negative mode were used for each metabolite. Derivatized short-chain fatty acid samples were separated with a Waters™ Atlantis dC18 column (100 Å, 3 µm, 3 mm × 100 mm) and eluted using a 6-min gradient from 5 to 80% B with buffer A as 0.1% formic acid in water and B as 0.1% formic acid in methanol. Short-chain fatty acids and central metabolic carboxylic acids were detected using MRMs from previously established methods, and identity was confirmed by comparison to derivatized standards^67,68^. All signals were integrated using MultiQuant® Software 3.0.3. Signals with greater than 50% missing values were discarded, and the remaining missing values were replaced with the lowest registered signal value. All signals with a QC coefficient of variance greater than 30% were discarded. Metabolites with multiple MRMs were quantified with the higher-intensity MRM. Filtered datasets were total sum normalized prior to analysis. Short-chain fatty acid datasets were stitched to their corresponding polar metabolite dataset via common signals for lactate. Single and multivariate analysis was performed in MarkerView® Software 1.3.1.

sPLSDA analyses of metabolomic data were performed in R using the mixOmics^70^ package and loadings and variates visualized in GraphPad Prism. Abundances of specific metabolites were visualized and analyzed in GraphPad Prism. Correlations between metabolites and bacterial genera were calculated and visualized in GraphPad Prism.

### Statistical analysis

Significance tests were performed as indicated where appropriate for the data using GraphPad Prism 9. Unless stated otherwise, statistical significance levels were determined as follows: ns = *p* > 0.05; * = *p* ≤ 0.05; ** = *p* ≤ 0.01; *** = *p* ≤ 0.001; **** = *p* ≤ 0.0001. The exact nature of tests is stated where appropriate.

## Supplementary Information

Supplementary Information

## Data Availability

All data are available on request from the corresponding authors or are uploaded to FigShare at 10.6084/m9.figshare.25796194.v1 and appropriate sequencing repositories (virome: PRJNA1076527, cytochrome b: PP212968, transcriptome: GSE254409). All material requests should be sent to V.J.M., vincent.munster@nih.gov.

## Code availability

All code is available upon request from J.R.P. Requests should be sent to juliarebecca.port@helmholtz-hzi.de.
