# Supplementary material for: Jamaican fruit bat (Artibeus jamaicensis) insusceptibility to mucosal inoculation with SARS-CoV-2 Delta variant is not caused by receptor compatibility
Source: Npj Viruses. 2024 Jul 16;2:26. doi: 10.1038/s44298-024-00037-1 (PMC11721433; doi:10.1038/s44298-024-00037-1)
Supplement: Supplementary file 1 — Supplementary Information [file 44298_2024_37_MOESM1_ESM.pdf]

Supplementary Information for

Jamaican fruit bat (*Artibeus jamaicensis*) insusceptibility to mucosal inoculation with SARS-CoV-2 Delta variant is not caused by receptor compatibility

Julia R. Port#[1], Jade C. Riopelle[1], Sarah van Tol[1], Arthur Wickenhagen[1], Eric Bohrnsen[2], Daniel E. Sturdevant[2], Rebecca Rosenke[2], Jamie Lovaglio[3], Justin Lack[2], Sarah L. Anzick[2], Kathleen Cordova[3], Kwe Claude Yinda[1], Patrick W. Hanley[3], Tony Schountz[4], Lon V. Kendall[4], Carl I. Shaia[3], Greg Saturday[3], Craig Martens[2], Benjamin Schwarz[2], Vincent J. Munster[1]

1. *Laboratory of Virology, Division of Intramural Research, National Institute of Allergy and Infectious Diseases, National Institutes of Health, Hamilton, MT, USA*

2. *Research Technologies Branch, Division of Intramural Research, National Institute of Allergy and Infectious Diseases, National Institutes of Health, Hamilton, MT, USA*

3. *Rocky Mountain Veterinary Branch, Division of Intramural Research, National Institute of Allergy and Infectious Diseases, National Institutes of Health, Hamilton, MT, USA*

4. *Department of Microbiology, Immunology, and Pathology, Colorado State University, Fort Collins, CO, USA*

# Corresponding author: juliarebecca.port@helmholtz-hzi.de

Author Contributions:

JRP designed the studies.

JRP, JCR, SvT, EB, JL, KC, KCY, PWH performed the experiments.

JRP, JCR, SvT, AW, DES, RR, JL, SA, KCY, CIS, GS, CM, BS analyzed results.

JRP, JCR, VJM wrote the manuscript.

All co-authors reviewed the manuscript.

Competing Interest Statement: No competing interests to disclose.

Keywords: bats, SARS-CoV-2, *Artibeus jamaicensis*, infection

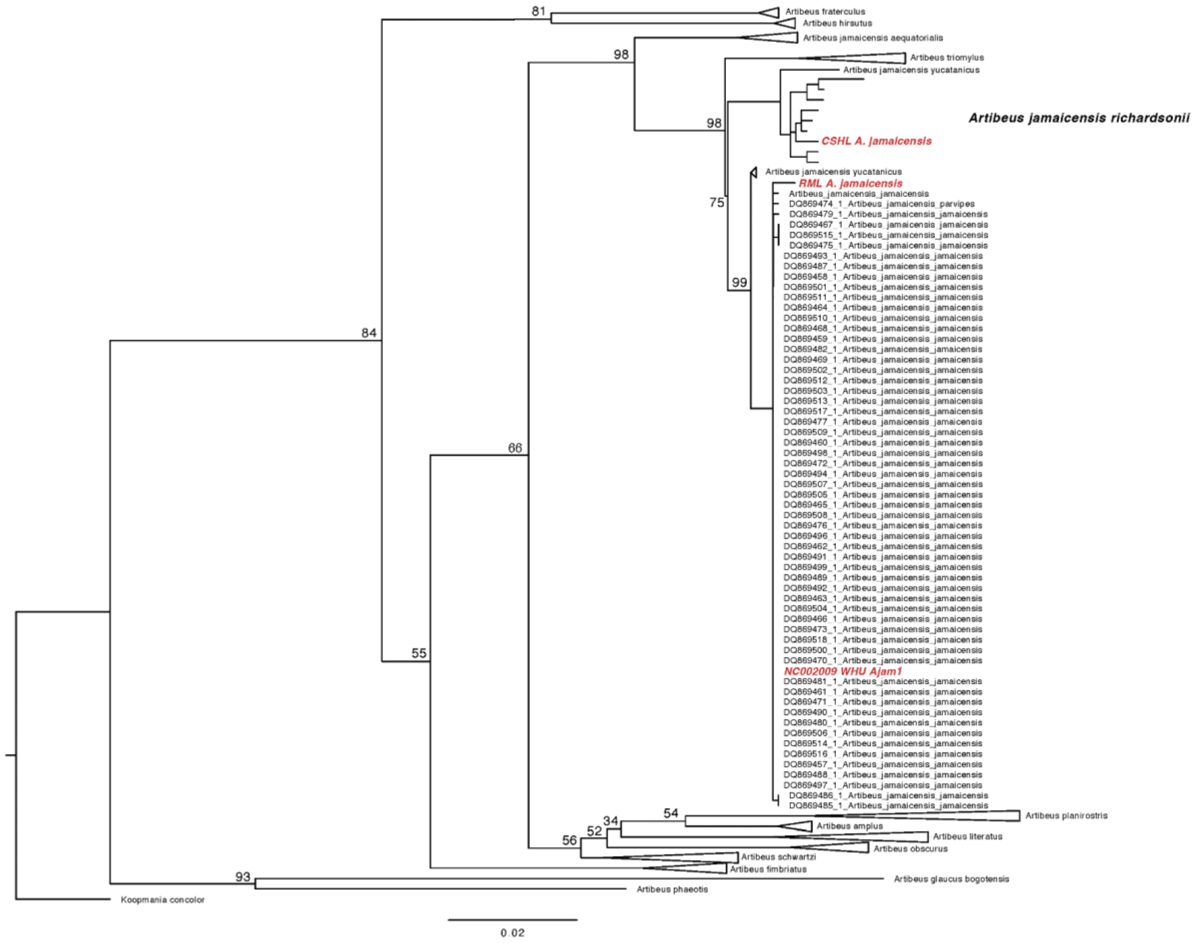

Supplementary Figure 1. Maximum likelihood phylogeny based on complete sequences of the Jamaican fruit bat mtDNA cytochrome b gene. Uninformative monophyletic nodes are collapsed to simplify the phylogeny, and all uncollapsed nodes are labelled with the bootstrap percentages.

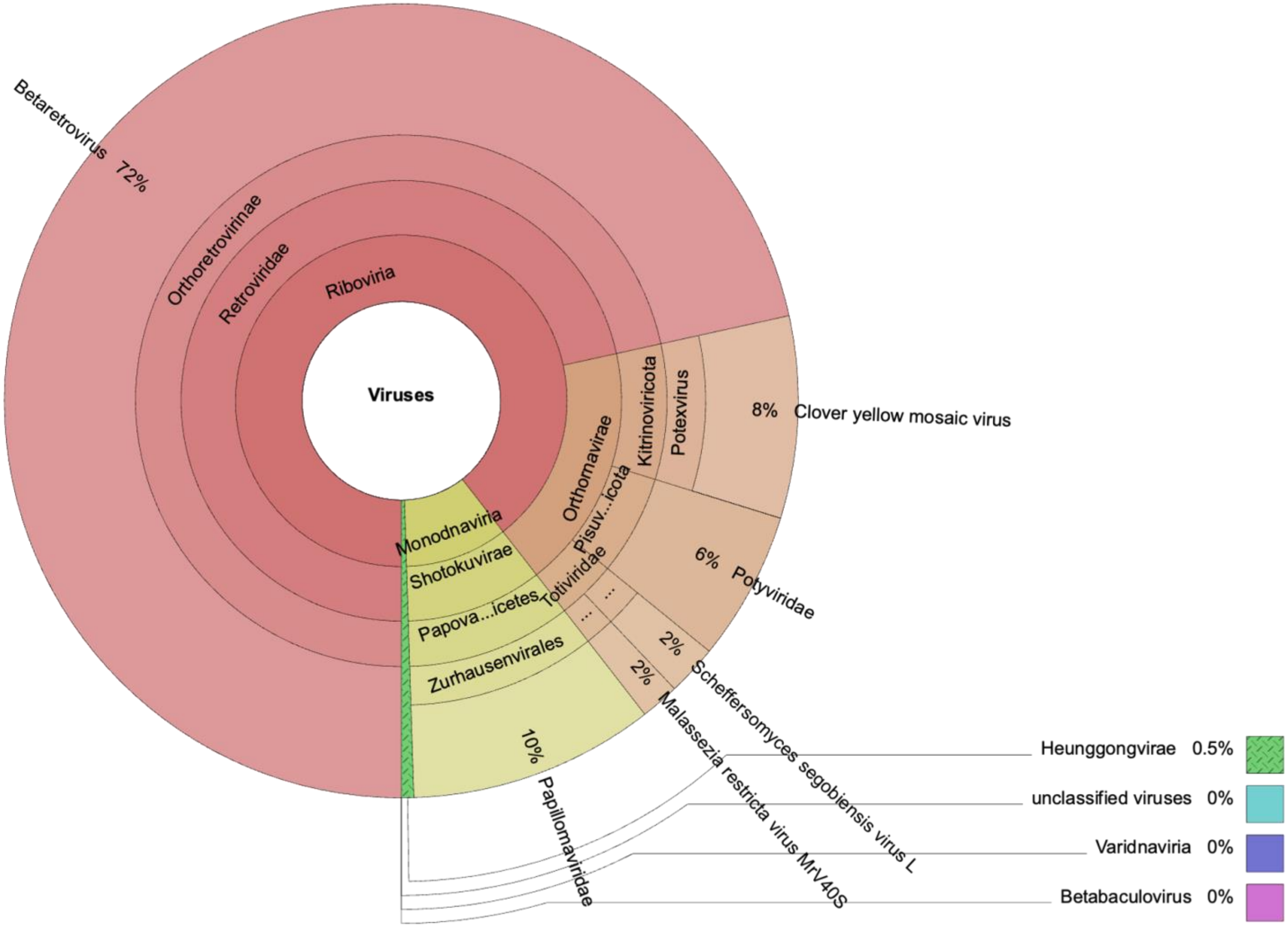

Supplementary Figure 2. Krona chart showing the virome of the captive Jamaican fruit bat colony.

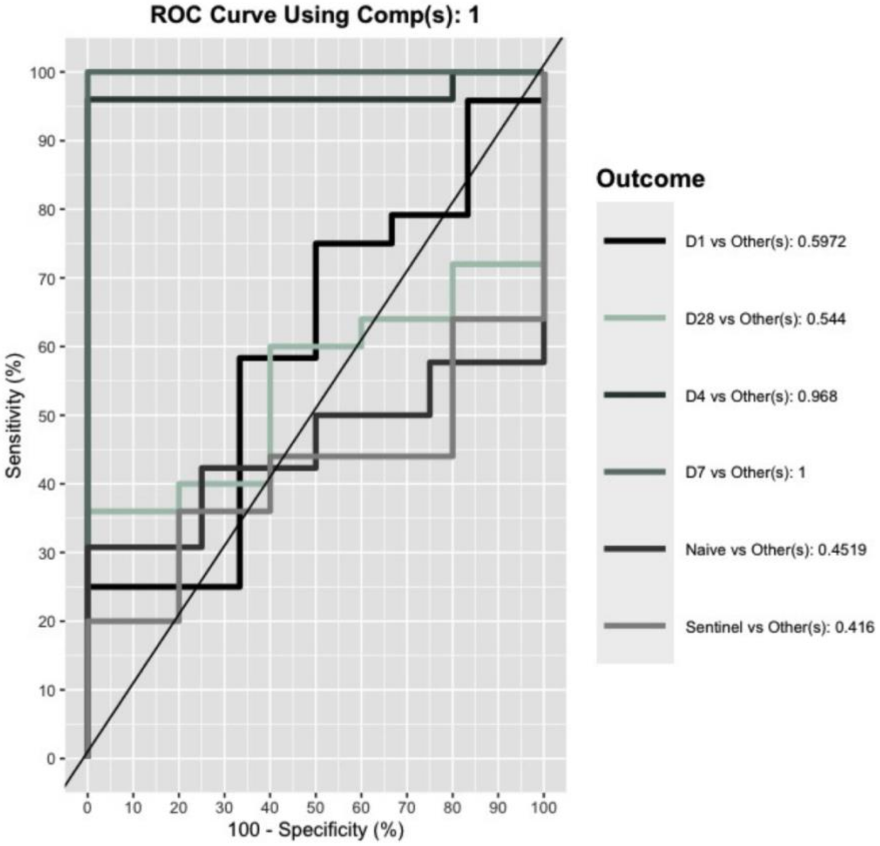

Supplementary Figure 3. Validation of sparse partial least squares-discriminant analysis of Jamaican fruit bat intestinal metabolome composition after inoculation with SARS-CoV-2 Delta variant. Receiver operating characteristic (ROC) curve for sPLSDA of time and inoculation status (N = 6 (1 DPI) / 5 (4, 7, 28 DPI, sentinels) / 4 (naïve animals)). Lines colored by group. Legend also shows the calculated area under the ROC curve (AUROC) for each predictor.

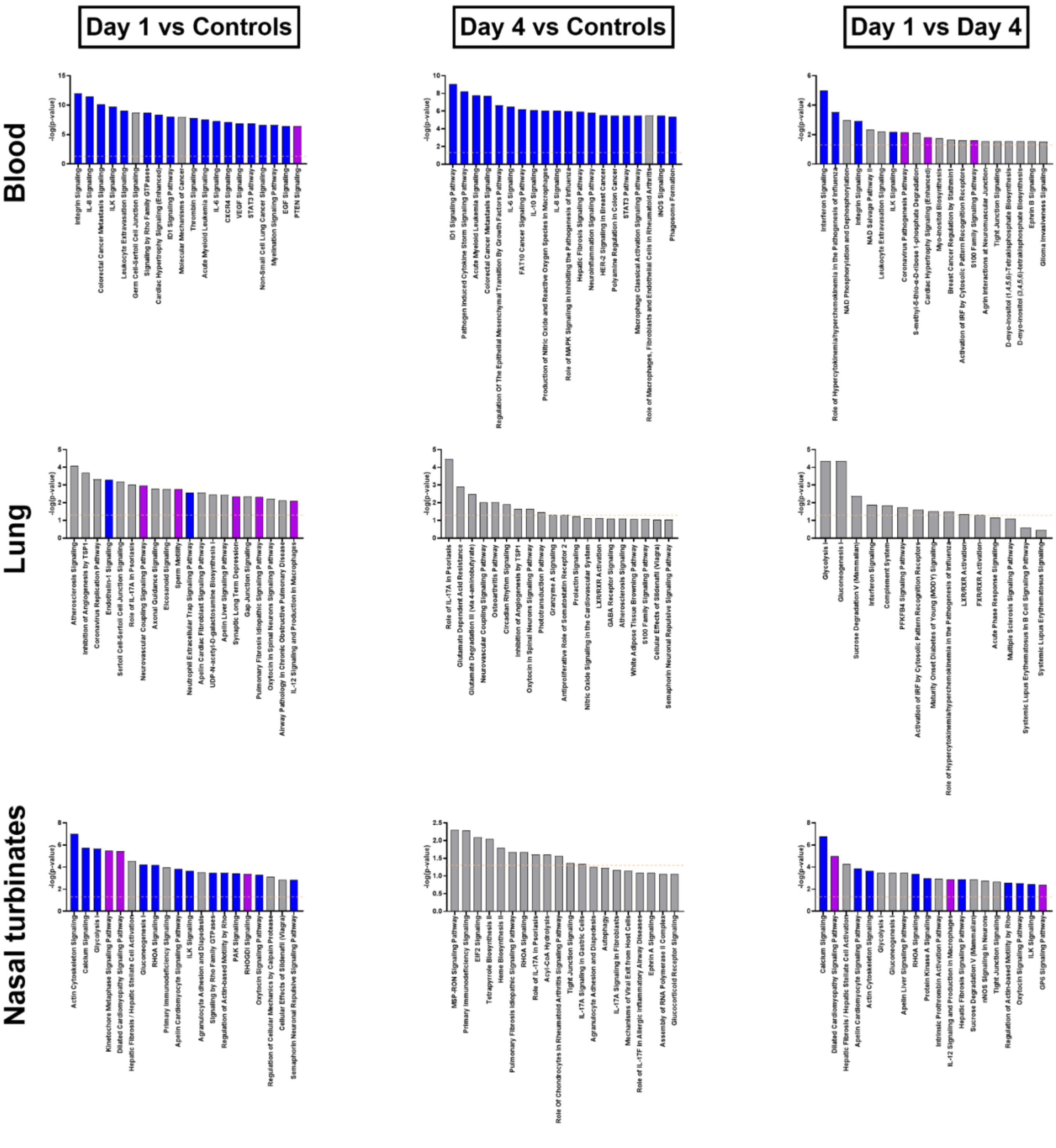

Supplementary Figure 4. Gene expression pathway up-or downregulation in Jamaican fruit bats after inoculation with SARS-CoV-2 Delta. Gene expression changes in blood, lungs, and nasal turbinates collected on day 1 and 4 after infection. Tissues collected from uninfected bats on day 28 served as comparison. Ingenuity Pathway Analysis of differentially expressed genes (DEGs) showing the top 20 pathways per comparison. Canonical pathways with a negative z-score are shown in blue, positive z-score in purple and pathways with an undefined or z-score = 0 are shown in grey. Significance threshold (p=0.05) is shown with a dotted orange line in each bar chart.

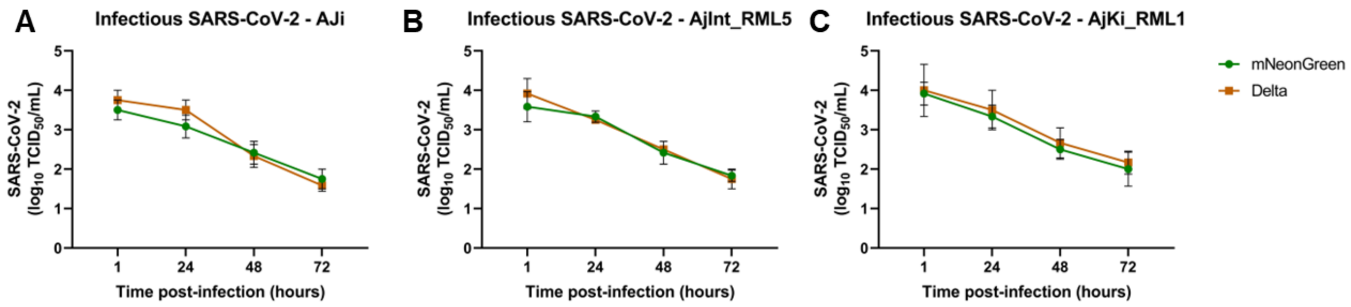

Supplementary Figure 5. SARS-CoV-2 replication is not supported in several Jamaican fruit bat cell lines. AJi (immortalized kidney), AjKi_RML1 (primary kidney), and AjInt_RML5 (primary intestinal) cells were infected with SARS-CoV-2 Delta or mNeonGreen at MOI 3.0. Supernatants were collected in triplicate to measure infectious virus.

Supplementary Table 1: Virome pipelines

Metavirs Pipeline

Megahit	
# CAT viral contigs	10
largest contig (bp)	3,647
# Kraken2 viral contigs	198
largest Kraken2 contig (bp)	11,071
Metaspades	
# CAT viral contigs	17
largest contig (bp)	3,944
# Kraken2 viral contigs	307
largest Kraken2 contig (bp)	5,311
# raw reads	2,345,827,700

Supplementary Table 2: Caging schematic

<table>
<tr><th></th><th></th><th></th><th></th><th>until D1</th><th>D1 -</th></tr>
<tr><th>Animal ID</th><th>Sex</th><th>Necropsy</th><th>Group</th><th>Cage #</th><th>Cage #</th></tr>
<tr><td>1</td><td>Female</td><td>1</td><td>D1</td><td rowspan="5">1</td><td rowspan="5">1</td></tr>
<tr><td>2</td><td>Female</td><td>1</td><td>D1</td></tr>
<tr><td>3</td><td>Female</td><td>1</td><td>D1</td></tr>
<tr><td>7</td><td>Female</td><td>4</td><td>D4</td></tr>
<tr><td>8</td><td>Female</td><td>4</td><td>D4</td></tr>
<tr><td>12</td><td>Female</td><td>7</td><td>D7</td><td rowspan="6">2</td><td rowspan="3">2</td></tr>
<tr><td>13</td><td>Female</td><td>7</td><td>D7</td></tr>
<tr><td>14</td><td>Female</td><td>7</td><td>D7</td></tr>
<tr><td>17</td><td>Female</td><td>28</td><td>D28</td><td rowspan="6">3</td></tr>
<tr><td>18</td><td>Female</td><td>28</td><td>D28</td></tr>
<tr><td>19</td><td>Female</td><td>28</td><td>D28</td></tr>
<tr><td>26</td><td>Female</td><td>28</td><td>sent</td><td rowspan="3">3</td></tr>
<tr><td>27</td><td>Female</td><td>28</td><td>sent</td></tr>
<tr><td>28</td><td>Female</td><td>28</td><td>sent</td></tr>
<tr><td>22</td><td>Female</td><td>28</td><td>control</td><td rowspan="2">7</td><td rowspan="2">7</td></tr>
<tr><td>23</td><td>Female</td><td>28</td><td>control</td></tr>
<tr><td>4</td><td>Female</td><td>1</td><td>D1</td><td rowspan="5">4</td><td rowspan="5">4</td></tr>
<tr><td>5</td><td>Female</td><td>1</td><td>D1</td></tr>
<tr><td>6</td><td>Female</td><td>4</td><td>D4</td></tr>
<tr><td>10</td><td>Female</td><td>4</td><td>D4</td></tr>
<tr><td>15</td><td>Female</td><td>7</td><td>D7</td></tr>
<tr><td>6</td><td>Male</td><td>1</td><td>D1</td><td rowspan="5">5</td><td rowspan="3">5</td></tr>
<tr><td>11</td><td>Male</td><td>4</td><td>D4</td></tr>
<tr><td>16</td><td>Male</td><td>7</td><td>D7</td></tr>
<tr><td>20</td><td>Male</td><td>28</td><td>D28</td><td rowspan="4">6</td></tr>
<tr><td>21</td><td>Male</td><td>28</td><td>D28</td></tr>
<tr><td>29</td><td>Male</td><td>28</td><td>sent</td><td rowspan="2">6</td></tr>
<tr><td>30</td><td>Male</td><td>28</td><td>sent</td></tr>
<tr><td>24</td><td>Male</td><td>28</td><td>control</td><td rowspan="2">8</td><td rowspan="2">8</td></tr>
<tr><td>25</td><td>Male</td><td>28</td><td>control</td></tr>
</table>

Supplementary Table 3: Serological status. Serological status was determined by A/G protein ELISA against the SARS-CoV-2 receptor binding domain. Virus neutralization (VN) titer was determined through neutralization of infectious virus.

**Animal ID**	Bat 1	Bat 2	Bat 3	Bat 4	Bat 5	Bat 6	Bat 7	Bat 8	Bat 9	Bat 10	Bat 11	Bat 12	Bat 13	Bat 14	Bat 15	Bat 16	Bat 17	Bat 18	Bat 19	Bat 20	Bat 21	Bat 26	Bat 27	Bat 28	Bat 29	Bat 30	Bat 22	Bat 23	Bat 24	Bat 25
**Group**	D1						D4					D7					D28					Sentinel					Control			
**Serological status**	Negative	Negative	Negative	Negative	Negative	Negative	Negative	Negative	Negative	Negative	Negative	Negative	Negative	Negative	Negative	Negative	Negative	Negative	Negative	Negative	Negative	Negative	Negative	Negative	Negative	Negative	Negative	Negative	Negative	Negative
**VN titer**	0	0	0	0	0	0	0	0	0	0	0	0	0	0	0	0	0	0	0	0	0	0	0	0	0	0	0	0	0	0

Supplementary Table 4: Pathological assessment. Histopathology was assessed by a board-certified pathologist. Lung score = interstitial infiltrate, liver = vascular change – glycogen type, kidney = interstitial fibrosis, lower GI = lymphoid patches: lymphocytolysis, bladder = submucosal lymphocytic infiltrate; 0 = no lesions, 1 =Minimal (1-10%), 2 = Mild (11-25%), 3 = Moderate (26-50%), 4 = Marked (51-75%), 5 = Severe (76-100%); np = no pathology assessed.

**Bat ID**	Bat 1	Bat 2	Bat 3	Bat 7	Bat 8	Bat 22	Bat 23	Bat 4	Bat 5	Bat 9	Bat 10	Bat 6	Bat 11	Bat 24	Bat 25
**Group**	d1	d1	d1	d4	d4	uninf	uninf	d1	d1	d4	d4	d1	d4	uninf	uninf
**Score**															
Lung	1	1	0	0	0	0	0	0	0	1	0	0	np	0	0
Trachea	0	np	0	0	0	0	0	0	0	np	np	0	np	0	0
Brain	0	0	0	0	0	0	0	0	0	0	0	0	np	0	0
Skull/nasal turbinates	0	0	0	0	0	0	0	0	0	0	0	0	np	0	0
Liver	4	3	3	3	3	3	4	3	3	3	3	3	np	3	3
Spleen	0	0	0	0	0	0	0	0	0	0	0	0	np	0	0
Kidney	0	0	0	0	0	0	0	0	0	0	0	0	np	0	1
Upper GI	0	0	0	0	0	0	0	0	0	0	0	0	np	0	0
Lower GI	0	0	0	0	0	0	0	0	0	2	0	0	np	3	3
Bladder	0	0	np	0	np	np	1	0	np	0	0	0	np	0	0
Heart	0	0	0	0	0	0	0	0	0	0	0	0		0	0
Radiograph lung score D0	0	0	0	0	0	np	np	0	0	0	0	0	0	np	np
Radiograph lung score D1 and D4	0	0	0	0	0	np	np	1	0	0	0	0	0	np	np
**SARS-CoV-2 IHC**															
Lung	0	0	0	np	np	np	np	0	0	np	np	np	np	np	0
Trachea	0	np	0	np	np	np	np	0	0	np	np	np	np	np	0
Brain	0	0	0	np	np	np	np	0	0	np	np	np	np	np	0
Skull	0	0	0	np	np	np	np	0	0	np	np	np	np	np	0
Liver	0	0	0	np	np	np	np	0	0	np	np	np	np	np	0
Spleen	0	0	0	np	np	np	np	0	0	np	np	np	np	np	0
Kidney	0	0	0	np	np	np	np	0	0	np	np	np	np	np	0
Upper GI	0	0	0	np	np	np	np	0	0	np	np	np	np	np	0
Lower GI	0	0	0	np	np	np	np	0	0	np	np	np	np	np	0
Urinary bladder	np	0	0	np	np	np	np	np	np	np	np	np	np	np	np
Ovary	0	0	0	np	np	np	np	np	np	np	np	np	np	np	np
Testicle	np	np	np	np	np	np	np	np	np	np	np	np	np	np	0
Heart	0	0	0	np	np	np	np	0	0	np	np	np	np	np	0

Supplementary Table 5: qRT measurements of tissues and swabs. UD = undetermined, no RNA copies detected, BQL = below detection limit of 10 copies/reaction. NT = nasal turbinates.

**Animal ID**	Bat 1	Bat 2	Bat 3	Bat 4	Bat 5	Bat 6	Bat 7	Bat 8	Bat 9	Bat 10	Bat 11	Bat 12	Bat 13	Bat 14	Bat 15	Bat 16	Bat 17	Bat 18	Bat 19	Bat 20	Bat 21	Bat 26	Bat 27	Bat 28	Bat 29	Bat 30	Bat 22	Bat 23	Bat 24	Bat 25
**Group**	D1						D4					D7					D28					Sentinel					Control			
**Oral Swab D0**	UD	UD	UD	UD	UD	UD	UD	UD	UD	UD	BQL	UD	UD	UD	UD	UD	BQL	UD	UD	UD	UD	UD	UD	UD	UD	UD	UD	UD	UD	UD
**Rectal Swab D0**	UD	UD	UD	UD	UD	UD	UD	UD	UD	UD	UD	UD	UD	BQL	UD	UD	UD	UD	BQL	UD	BQL	UD	UD	UD	UD	UD	UD	UD	UD	UD
**Oral Swab D1**	UD	UD	UD	UD	UD	UD	UD	UD	UD	UD	UD	UD	BQL	UD	UD	UD	UD	UD	UD	UD	UD	UD	UD	UD	UD	UD	UD	UD	UD	UD
**Rectal Swab D1**	UD	UD	UD	UD	UD	UD	UD	UD	UD	UD	UD	UD	UD	UD	UD	UD	UD	UD	UD	UD	UD	UD	UD	UD	UD	UD	UD	UD	UD	UD
**Oral Swab D2**							UD	UD	UD	UD	UD	UD	UD	UD	BQL	UD	UD	UD	UD	UD	UD	UD	UD	UD	UD	UD	UD	UD	UD	UD
**Rectal Swab D2**							UD	UD	UD	UD	UD	UD	UD	UD	UD	UD	UD	UD	UD	UD	UD	UD	UD	UD	UD	UD	UD	UD	UD	UD
**Oral Swab D3**							UD	UD	UD	UD	UD	UD	UD	UD	UD	UD	UD	UD	UD	UD	UD	UD	UD	UD	UD	UD	UD	UD	UD	UD
**Rectal Swab D3**							UD	UD	UD	UD	UD	UD	UD	UD	UD	UD	UD	UD	UD	UD	UD	UD	UD	UD	UD	UD	UD	UD	UD	UD
**Oral Swab D4**							UD	UD	UD	UD	UD	UD	UD	UD	UD	UD	UD	UD	UD	UD	UD	UD	UD	UD	UD	UD	UD	UD	UD	UD
**Rectal Swab D4**							UD	UD	UD	UD	UD	UD	UD	UD	UD	UD	UD	UD	UD	UD	UD	UD	UD	UD	UD	UD	UD	UD	UD	UD
**Oral Swab D7**												UD	UD	UD	UD	UD	UD	UD	UD	UD	UD	UD	UD	UD	UD	UD	UD	UD	UD	UD
**Rectal Swab D7**												UD	UD	UD	UD	UD	UD	UD	UD	UD	UD	UD	UD	UD	UD	UD	UD	UD	UD	UD
**Bladder**	UD	UD	UD	UD	UD	UD	UD	UD	UD	UD	UD	UD	UD	UD	UD	UD														
**Brain**	UD	UD	UD	UD	UD	UD	UD	UD	UD	UD	UD	UD	UD	UD	UD	UD														
**Intestine**	UD	UD	UD	UD	UD	UD	UD	UD	UD	UD	UD	UD	UD	UD	UD	UD														
**Kidney**	UD	UD	UD	UD	UD	UD	UD	UD	UD	UD	UD	UD	UD	UD	UD	UD														
**Liver**	UD	UD	UD	UD	UD	UD	UD	UD	UD	UD	UD	UD	UD	UD	UD	UD														
**Lung**	UD	8E+06	1.783	UD	UD	UD	UD	UD	UD	UD	UD	UD	UD	UD	UD	UD														
**NT**	UD	UD	UD	UD	UD	UD	UD	UD	UD	UD	UD	UD	UD	UD	UD	UD														

Supplementary Table 6: Number of up- or down regulated genes in Jamaican fruit bats after inoculation with Delta.

	**Blood**			**Lungs**			**Nasal Turbinates**		
**Expression**	Day 1 vs Controls	Day 4 vs Controls	Day 1 vs Day 4	Day 1 vs Controls	Day 4 vs Controls	Day 1 vs Day 4	Day 1 vs Controls	Day 4 vs Controls	Day 1 vs Day 4
**Upregulated**	839	358	22	62	12	11	60	11	79
**Downregulated**	1079	545	124	61	10	3	180	57	69
**Unchanged**	19870	20885	21642	21665	21766	21774	21548	21720	21640

Supplementary Table 7: Top 10 significantly up- or down-regulated genes in Jamaican fruit bats after inoculation with Delta

**Blood**					**Lung**					**Nasal Turbinates**				
Day 1 vs. Day 4					***Day 1 vs. Day 5***					***Day 1 vs. Day 6***				
**Gene name**	**log2FoldChan**	**pvalue**	**padj**	**Expression**	**Gene name**	**log2FoldChan**	**pvalu**	**padj**	**Expression**	**Gene name**	**log2FoldChan**	**pvalue**	**padj**	**Expression**
CMYA5	-16.78	2.94E-	3.29E-19	Down-	LOC11905473	28.61	1.79E-	3.43E-21	Up-regulated	TNNT3	-4.41	1.14E-	2.15E-05	Down-
KLHL41	-16.26	5.84E-	3.27E-15	Down-	RETNLB	26.56	7.60E-	7.29E-08	Up-regulated	MYBPC1	-4.55	1.81E-	0.00016980	Down-
LOC11904718	2.92	3.77E-	1.41E-09	Up-regulated	BPIFA2	21.29	1.30E-	8.29E-08	Up-regulated	MYL4	-5.21	1.79E-	0.00111224	Down-
IFI6	-4.77	2.33E-	6.52E-05	Down-	LOC11905478	15.43	4.65E-	2.23E-06	Up-regulated	NRIP3	3.51	2.37E-	0.00111224	Up-regulated
ACBD6	-1.60	4.62E-	9.74E-05	Down-	FBP2	-16.11	2.02E-	7.76E-06	Down-	HK2	-3.16	3.20E-	0.00119931	Down-
IFIT3	-2.75	5.21E-	9.74E-05	Down-	ALDOB	4.45	1.25E-	0.00399963	Up-regulated	CAPN3	-4.30	8.36E-	0.00247423	Down-
PLN	-5.72	1.03E-	0.00016520	Down-	LOC11905820	13.76	2.42E-	0.00662599	Up-regulated	CPNE6	5.01	1.06E-	0.00247423	Up-regulated
CENPV	-2.11	1.95E-	0.00027351	Down-	GSDMC	-5.02	3.37E-	0.00717081	Down-	PHF14	2.36	9.83E-	0.00247423	Up-regulated
OAS1	-3.81	6.26E-	0.00077906	Down-	LOC11904158	4.13	3.03E-	0.00717081	Up-regulated	MS4A15	5.21	1.22E-	0.00254818	Up-regulated
DTX3L	-1.48	8.07E-	0.00090414	Down-	ISG15	-2.85	8.29E-	0.01476379	Down-	TRIM63	-4.11	2.21E-	0.00413702	Down-
Day 1 vs. Controls					***Day 1 vs. Controls***					***Day 1 vs. Controls***				
**Gene name**	**log2FoldChan**	**pvalue**	**padj**	**Expression**	**Gene name**	**log2FoldChan**	**pvalu**	**padj**	**Expression**	**Gene name**	**log2FoldChan**	**pvalue**	**padj**	**Expression**
CKMT2	-19.54	2.73E-	4.32E-20	Down-	LOC11905473	29.68	1.25E-	1.81E-21	Up-regulated	ABRA	-46.07	######	7.01E-107	Down-
CAV3	-17.11	3.78E-	2.99E-15	Down-	ACE2	8.17	5.09E-	0.00037068	Up-regulated	ACTA1	-9.85	1.18E-	1.06E-10	Down-
TRIM54	-16.67	3.14E-	1.66E-14	Down-	COL12A1	2.19	8.78E-	0.00039946	Up-regulated	TNNT3	-5.31	5.96E-	3.56E-08	Down-
SKAP2	-1.70	5.58E-	2.21E-14	Down-	GGT7	-1.45	1.64E-	0.00039946	Down-	ATP2A1	-7.23	8.91E-	3.99E-08	Down-
FITM1	-15.64	2.19E-	6.93E-11	Down-	LOC11905820	15.64	2.18E-	0.00039946	Up-regulated	PGAM2	-8.33	2.10E-	6.27E-07	Down-
RNF130	-2.02	2.76E-	7.29E-11	Down-	MORN1	-1.77	2.32E-	0.00039946	Down-	SMPX	-8.41	1.93E-	6.27E-07	Down-
PYGL	-2.35	3.33E-	7.54E-11	Down-	NDUFA4L2	6.18	2.39E-	0.00039946	Up-regulated	CAPN3	-6.05	4.75E-	1.06E-06	Down-
C5AR1	-2.64	7.31E-	1.20E-10	Down-	SEPTIN11	0.91	2.47E-	0.00039946	Up-regulated	CMYA5	-7.78	4.74E-	1.06E-06	Down-
LOC11906530	12.69	7.10E-	1.20E-10	Up-regulated	TST	-1.65	1.68E-	0.00039946	Down-	APOBEC2	-8.16	7.43E-	1.48E-06	Down-
NUMB	-2.05	7.56E-	1.20E-10	Down-	GUCY1B1	2.51	5.27E-	0.00076716	Up-regulated	LOC11904576	-6.04	1.77E-	3.17E-06	Down-
Day 4 vs. Controls					***Day 4 vs. Controls***					***Day 4 vs. Controls***				
**Gene name**	**log2FoldChan**	**pvalue**	**padj**	**Expression**	**Gene name**	**log2FoldChan**	**pvalu**	**padj**	**Expression**	**Gene name**	**log2FoldChan**	**pvalue**	**padj**	**Expression**
GALNT13	42.08	1.62E-	2.43E-104	Up-regulated	BPIFA2	-21.21	1.15E-	2.02E-05	Down-	ABRA	-40.37	3.25E-	5.83E-74	Down-
SLC34A1	-39.95	4.09E-	3.06E-49	Down-	RETNLB	-24.10	2.08E-	0.00018227	Down-	LOC11905820	18.00	5.97E-	5.35E-05	Up-regulated
CMYA5	16.44	1.53E-	7.65E-15	Up-regulated	NDUFA4L2	7.47	3.40E-	0.00019825	Up-regulated	LOC11906418	-6.50	7.83E-	0.00046754	Down-
KLHL41	16.47	2.41E-	9.04E-14	Up-regulated	PLEKHG1	1.06	5.20E-	0.00227453	Up-regulated	LOC11905295	-9.51	5.19E-	0.00186128	Down-
ACSS2	-2.18	1.58E-	3.95E-12	Down-	CXCL13	-5.50	1.85E-	0.00646980	Down-	LOC11905447	-5.11	5.08E-	0.00186128	Down-
GK	-2.51	1.45E-	3.95E-12	Down-	SAMD5	3.11	1.00E-	0.02502378	Up-regulated	SFTPC	-5.86	1.01E-	0.00302374	Down-
PDK3	-2.04	2.70E-	5.77E-12	Down-	SPARCL1	1.40	9.03E-	0.02502378	Up-regulated	KIF15	1.95	2.71E-	0.00694808	Up-regulated
NUMB	-2.11	1.47E-	2.75E-11	Down-	TRPC6	2.12	1.53E-	0.03337066	Up-regulated	LOC11904491	-6.07	6.87E-	0.01375686	Down-
FITM1	-16.05	1.89E-	3.15E-11	Down-	IBA57	-0.88	1.86E-	0.03626525	Down-	RAG1	6.78	7.68E-	0.01375686	Up-regulated
TRIM54	-15.19	5.14E-	7.70E-11	Down-	ANK3	0.95	3.35E-	0.04213147	Up-regulated	RAG2	6.12	7.52E-	0.01375686	Up-regulated
